# Inhibition of Translation Initiation by Protein 169: A Vaccinia Virus Strategy to Suppress Innate and Adaptive Immunity and Alter Virus Virulence

**DOI:** 10.1371/journal.ppat.1005151

**Published:** 2015-09-03

**Authors:** Pavla Strnadova, Hongwei Ren, Robert Valentine, Michela Mazzon, Trevor R. Sweeney, Ian Brierley, Geoffrey L. Smith

**Affiliations:** 1 Department of Pathology, University of Cambridge, Cambridge, United Kingdom; 2 Department of Virology, Faculty of Medicine, Imperial College London, London, United Kingdom; McMaster University, CANADA

## Abstract

Vaccinia virus (VACV) is the prototypic orthopoxvirus and the vaccine used to eradicate smallpox. Here we show that VACV strain Western Reserve protein 169 is a cytoplasmic polypeptide expressed early during infection that is excluded from virus factories and inhibits the initiation of cap-dependent and cap-independent translation. Ectopic expression of protein 169 causes the accumulation of 80S ribosomes, a reduction of polysomes, and inhibition of protein expression deriving from activation of multiple innate immune signaling pathways. A virus lacking 169 (vΔ169) replicates and spreads normally in cell culture but is more virulent than parental and revertant control viruses in intranasal and intradermal murine models of infection. Intranasal infection by vΔ169 caused increased pro-inflammatory cytokines and chemokines, infiltration of pulmonary leukocytes, and lung weight. These alterations in innate immunity resulted in a stronger CD8^+^ T-cell memory response and better protection against virus challenge. This work illustrates how inhibition of host protein synthesis can be a strategy for virus suppression of innate and adaptive immunity.

## Author Summary

Long after smallpox was eradicated by vaccination with vaccinia virus, the study of this virus continues to reveal novel aspects of the interactions between a virus and the host in which it replicates. In this work we investigated the function of a previously uncharacterized VACV protein, called 169. The results show that protein 169 inhibits the synthesis of host proteins in cells and thereby provides a broad inhibition of the host innate immune response to infection. Unlike several other virus inhibitors of host protein synthesis, protein 169 acts by inhibiting the initiation of protein synthesis by both cap-dependent and cap-independent pathways. Also unlike several other virus protein synthesis inhibitors, the loss of protein 169 does not affect virus replication or spread, but the virus virulence was increased. This more severe infection is, however, cleared more rapidly and results in a stronger immunological memory response that is mediated by T-cells and provides better protection against re-infection. This work illustrates how shutting down host protein synthesis can be a strategy to block the host immune response to infection rather than a means to manufacture more virus particles.

## Introduction

The study of virus-host interactions continues to provide valuable information about the complex relationships between cells and pathogens. Large DNA viruses, in particular, encode many proteins that modify the intracellular environment to promote viral survival, replication and spread. *Vaccinia virus* (VACV) is the prototypic *Orthopoxvirus* of the *Poxviridae* and is the vaccine used to eradicate smallpox [1]. VACV replicates in the cytoplasm and encodes about 200 proteins that are required for viral transcription and replication [2, 3], alteration of cell metabolism [4–7], and immune evasion [8].

Between one-third and one-half of VACV proteins are devoted to evasion of innate immunity and these immunevasins may function inside or outside the infected cell. Intracellular immunevasins include those that inhibit innate immune signaling pathways leading to activation of nuclear factor kappa-light-chain-enhancer of activated B cells (NF-κB), interferon (IFN) regulatory factor (IRF)-3 and Janus kinase (JAK) / signal transducer and activation of transcription (STAT) signaling. Other intracellular immunevasins suppress apoptosis or the antiviral activity of IFN-stimulated gene products. Additional immunevasins are secreted from infected cells to bind complement factors, IFNs, cytokines or chemokines extracellularly and inhibit their activity. An interesting aspect of these immune evasion strategies is the apparent redundancy, with several proteins targeting the same activation pathway. For instance, there are at least 10 intracellular inhibitors of NF-κB encoded by VACV [9–18] and a VACV strain lacking all these factors still inhibits NF-κB [19].

VACV, like all viruses, relies on host ribosomes for virus protein synthesis. To ensure efficient translation of virus proteins, VACV shuts off host protein synthesis and re-directs the cellular translational machinery to the synthesis of viral proteins [20–27]. VACV mRNAs are translated by a cap-dependent mechanism facilitated by the eukaryotic initiation factor (eIF)4F complex that recognizes the 5’-methylated cap, and translation is initiated by interaction of the cap with eIF4E, a cap-binding protein [28]. VACV encodes capping [29] and methylating enzymes [30] that produce viral mRNAs that mimic cellular mRNAs and so evade detection by host pattern recognition receptors. VACV protein synthesis occurs in virus factories [21, 27, 31], and to ensure preferential translation of virus mRNAs, VACV expresses de-capping enzymes D9 and D10 that remove the cap from both cellular and viral mRNAs [25, 32, 33]. The abundance of viral transcripts ensures translation of viral mRNA continues despite this de-capping activity, which also promotes turnover of viral mRNAs and thereby aids the transition between the early, intermediate and late stages of viral gene expression. The importance of protein D10 for the virus replication cycle is illustrated by a D10 deletion mutant that has a smaller plaque phenotype and produces reduced yields of virus in cell culture [26]. Moreover, mutant viruses with a stop codon introduced into the D10 open reading frame (ORF) or with amino acid alterations in the D10 catalytic site have an attenuated phenotype *in vivo* [34]. D9 and D10 also reduce dsRNA accumulation and the consequential activation of host responses [35]. A similar outcome was observed after VACV infection of cells lacking the host exonuclease Xrn1 [36].

This report presents a functional characterization of VACV strain Western Reserve (WR) protein 169, a previously uncharacterized protein that is expressed by some, but not all VACV strains and orthopoxviruses. Protein 169 is an inhibitor of cap-dependent and cap-independent translational initiation. Protein 169 localizes in cytoplasmic puncta and is largely excluded from virus factories, enabling preferential inhibition of host mRNA translation. Consistent with this, protein 169 does not affect virus replication or spread in cell culture, but is a potent inhibitor of translation in cells in which it is expressed ectopically. Consequently, protein 169 blocks expression of host proteins that are induced following activation of diverse innate immune signaling pathways, and, in two *in vivo* models of VACV infection, a virus lacking 169 (vΔ169) induces a more severe primary infection than control viruses. The altered disease severity is not due to changes in viral replication, but instead is associated with increased production of pro-inflammatory cytokines and chemokines, and increased recruitment of immune cells at the site of infection. This altered response also affects the adaptive memory response and causes increased CD8^+^ T-cell memory and better protection against virus challenge.

Collectively, these results indicate that virus inhibition of host protein synthesis can be a strategy to suppress innate and adaptive immunity, rather than primarily a means to aid virus replication as considered hitherto.

## Results

### Characterization of the VACV 169 protein

VACV strain WR gene *169R* encodes a small, charged protein of 78 amino acid residues. The protein lacks a nuclear localisation signal and a hydrophobic transmembrane sequence suggesting that protein 169 is likely to be cytosolic. The ORF is conserved in VACV strains modified vaccinia virus Ankara (MVA), Lister, Duke, Acambis 3000 and rabbitpox virus, and other orthopoxviruses such as camelpox virus, taterapox virus, cowpox virus and monkeypox virus (S1 Fig). However, the ORF is truncated in multiple variola virus strains (the cause of smallpox) after codon 38 and in ectromelia virus (ECTV) after codon 41. In cowpox virus and monkeypox virus there are minor changes in amino acid length and composition, but the protein is identical in the VACV strains shown and in taterapox virus (S1 Fig). The truncation of this ORF in VACV strain Copenhagen and in other orthopoxviruses indicates that the 78 amino acid protein is non-essential for orthopoxvirus replication.

**Fig 1:**
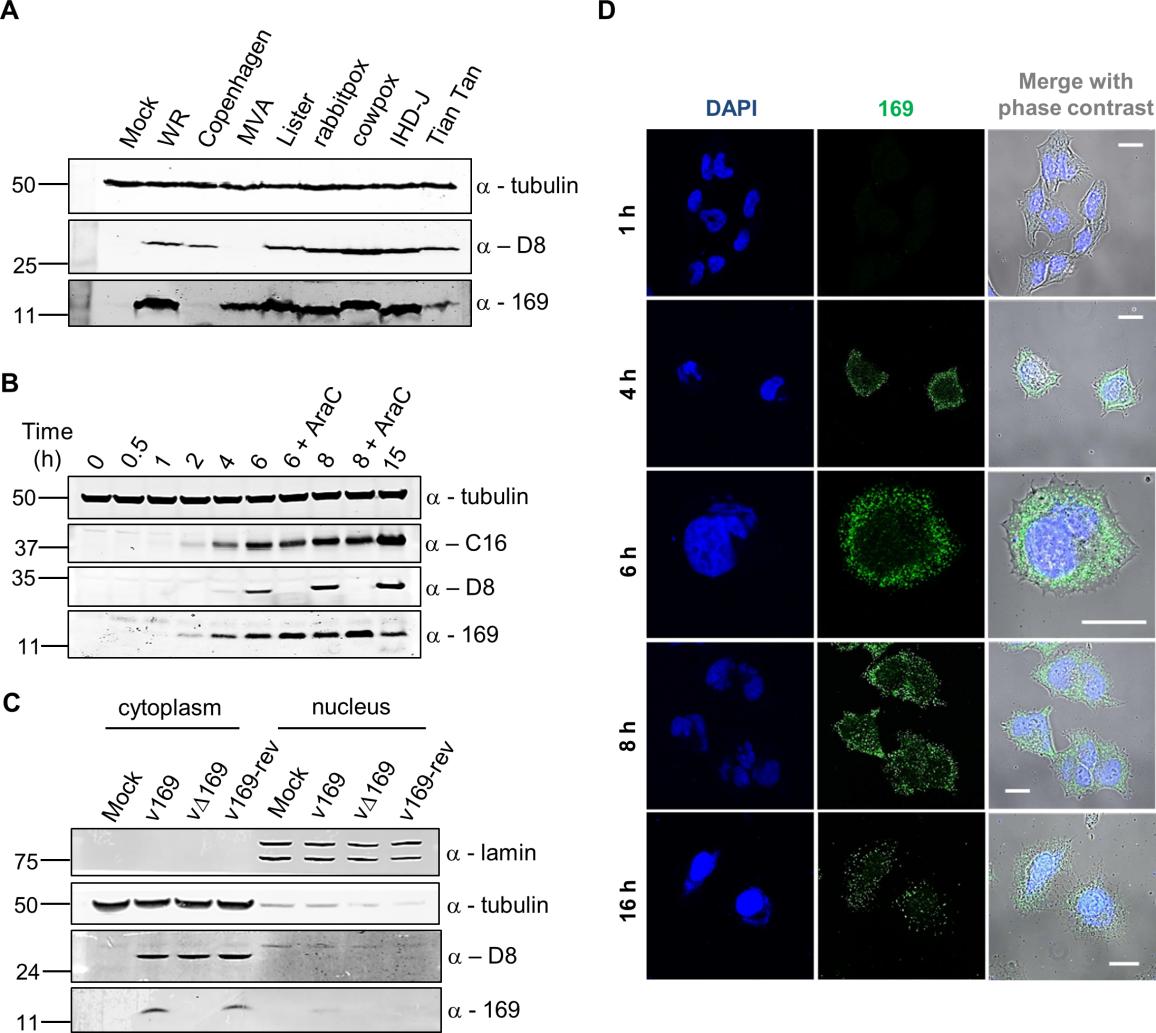
Protein 169 is expressed early and localizes in cytoplasmic puncta. (**A**) HeLa cells were either mock-infected or infected at 5 plaque-forming units (PFU)/cell with different VACV strains or cowpox virus. Cell lysates were prepared 16 h p.i., separated by SDS-PAGE and immunoblotted using antibodies against 169, VACV protein D8 or α-tubulin. (**B**) HeLa cells were infected with v169 at 10 PFU/cell in the presence or absence of AraC (40 μg/ml) for the indicated times. Cell lysates were resolved by SDS-PAGE and immunoblotted using the indicated antibodies. (**C**) HeLa cells were either mock-infected or infected with indicated viruses at 10 PFU/cell. After 7 h, cells were separated into nuclear and cytoplasmic fractions and analysed by SDS-PAGE and immunoblotting with the indicated antibodies. In all immunoblots, molecular size markers are indicated on the left in kDa. (**D**) HeLa cells were infected with v169 at 10 PFU/cell for the indicated times and at 2 PFU/cell for 16 h. Cells were fixed and stained with anti-169 purified antibody (green) and DAPI (blue). White lines are scale bars (20 μm).

The expression of protein 169 by several VACV strains was investigated by immunoblotting using a rabbit polyclonal antibody raised against VACV WR protein 169 that had been expressed in and purified from *E*. *coli* (Methods). This detected a 13-kDa polypeptide in cells infected with VACV strains WR, MVA, Lister, rabbitpox, International Health Department (IHD)-J and Tian Tan, and cowpox virus strain Brighton Red, but not VACV strain Copenhagen, or in mock-infected cells (Fig 1A). VACV infection was confirmed by immunoblotting with a mAb that recognizes the VACV structural protein D8 [37], although this mAb did not detect the D8 protein made by MVA (Fig 1A). Immunoblotting for α-tubulin demonstrated equal loading of samples.

### Protein 169 is expressed early and localizes in the cytoplasm

The time of expression and localization of protein 169 during infection were investigated by immunoblotting (Fig 1B and 1C) and immunofluorescence microscopy (Fig 1D). HeLa cells were infected with v169 (a plaque purified, wild-type virus that expresses protein 169) in the presence or absence of cytosine arabinoside (AraC), a DNA replication inhibitor that blocks intermediate and late VACV gene expression. The anti-169 antiserum detected a 13-kDa protein from 2 h p.i. that was also present following addition of AraC, showing expression prior to DNA replication (Fig 1B). Similar expression kinetics were observed for early VACV protein C16 [38]. In contrast, the VACV late protein D8 [39] was expressed only in the absence of AraC.

The localization of protein 169 was investigated by biochemical fractionation of infected cells. Immunoblotting of lysates from cells infected with v169, vΔ169 (a deletion mutant lacking the *169R* gene) and v169-rev (a revertant virus in which the *169R* gene was reinserted at its natural locus into vΔ169) showed that protein 169 is expressed from v169 and v169-rev, but not vΔ169, and that it localizes predominantly in the cytoplasm. Satisfactory separation of cytoplasmic and nuclear fractions was confirmed by blotting for α-tubulin and lamin (Fig 1C). Analysis by immunofluorescence using purified anti-169 antibody (Methods) detected protein 169 from 4 h p.i. in cytoplasmic puncta (Fig 1D). VACV factories were also detected from 4 h p.i. by DAPI staining, but protein 169 was excluded from these structures.

**Fig 2:**
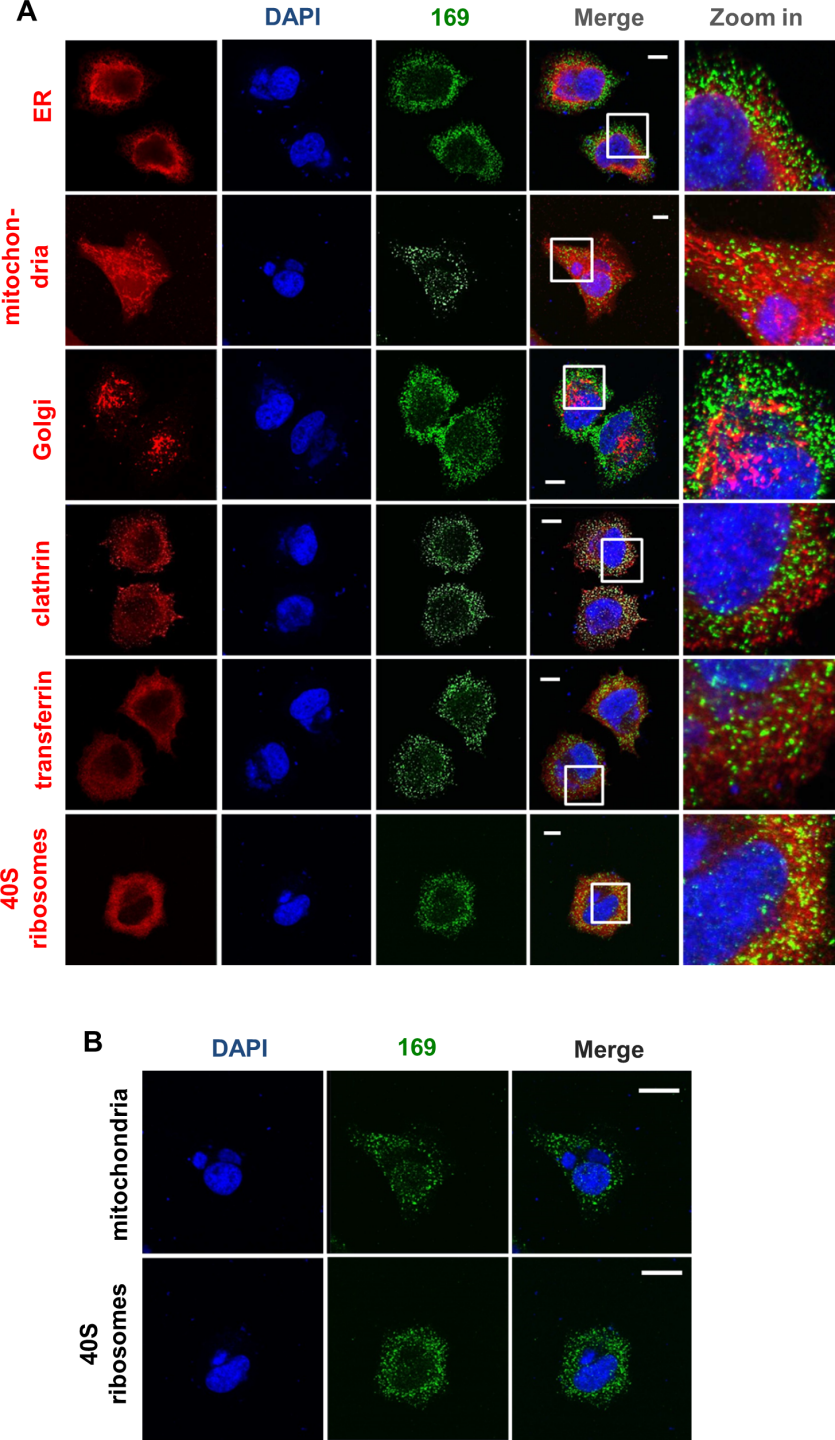
Protein 169 is present in cytoplasmic puncta and is excluded from factories. (**A**) HeLa cells were infected with v169 at 10 PFU/per cell for 7 h before cells were fixed and stained with anti-169 purified antibody (green), DAPI (blue) and with antibodies against markers of different intracellular compartments (red): protein disulphide isomerase (PDI) for endoplasmic reticulum (ER), GM130 for Golgi apparatus (Golgi), clathrin for some membrane vesicles, transferrin for endosomes and protein S6 for 40S ribosomes. Mitochondria were stained by addition of mitotracker to live cells that were then fixed and stained with anti-169 and with DAPI. White lines are scale bars (10 μm). (**B**) Enlarged versions of the mitochondrial and 40S ribosome staining from panel A showing DAPI-stained nuclei and virus factories (blue) and protein 169 (green). White lines are scale bars (20 μm).

To determine if protein 169 co-localized with specific cytoplasmic organelles, infected cells were stained with antibodies that detected the endoplasmic reticulum, mitochondria, Golgi apparatus, clathrin-containing vesicles and endosomes but no clear co-localization was observed (Fig 2A). Partial co-localization with 40S ribosomes was noted, although the abundance of 40S ribosomes makes a clear correlation uncertain. Staining with DAPI confirmed that protein 169 was excluded from virus factories (Fig 2B).

### 169 is not required for viral replication and spread *in vitro*

**Fig 3:**
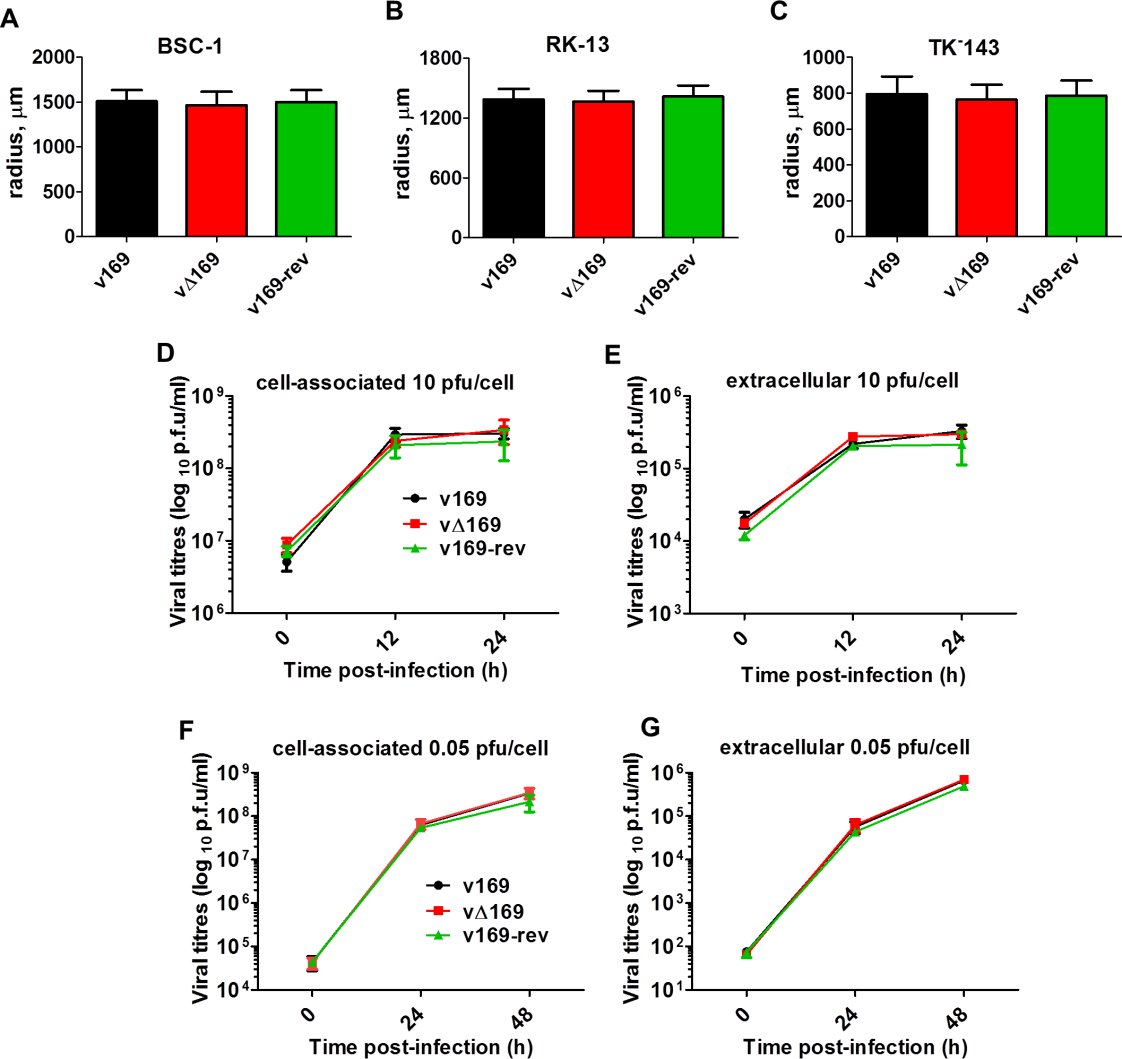
Protein 169 does not affect virus replication or spread *in vitro*. (**A-C**) BSC-1, TK^-^143 and RK-13 cells were infected with the indicated viruses at 50 PFU/well of a 6-well plate. After 72 h, cells were stained with crystal violet and the radius of plaques (*n* = 20 per virus) was measured on a Zeiss Axio Vert.A1 microscope with AxioCam MRc and Axiovision 4.8 software. Results are expressed as the mean of radii of plaque size ± SD. Data shown are from one representative experiment (*n* = 3). (**D**, **E**) BSC-1 cells were infected at 10 PFU/cell and the amount of cell-associated virus (D) and extracellular virus (E) was determined at different times p.i. by plaque assay. (**F**, **G**) As in (D, E) except that cells were infected at 0.05 PFU/cell. Results are expressed as the mean titer per sample ± SD log_10_ PFU. Data from one representative experiment (*n* = 2) are shown.

The contribution of protein 169 to virus replication and spread was investigated using recombinant VACVs v169, vΔ169, and v169-rev that were constructed by transient dominant selection [40] (Methods). These three viruses formed plaques of indistinguishable size in African monkey fibroblasts (BSC-1) and also in rabbit kidney (RK)-13 cells and human TK^-^143 cells (Fig 3A–3C). Similarly, the yields of intracellular and extracellular vΔ169 were unaltered compared to control viruses after high (10 PFU/cell) or low (0.05 PFU/cell) multiplicity of infection in BSC-1 cells (Fig 3D–3G). Therefore, the 169 protein is non-essential for virus replication and spread in cell culture.

### 169 inhibits various signaling pathways at protein level

**Fig 4:**
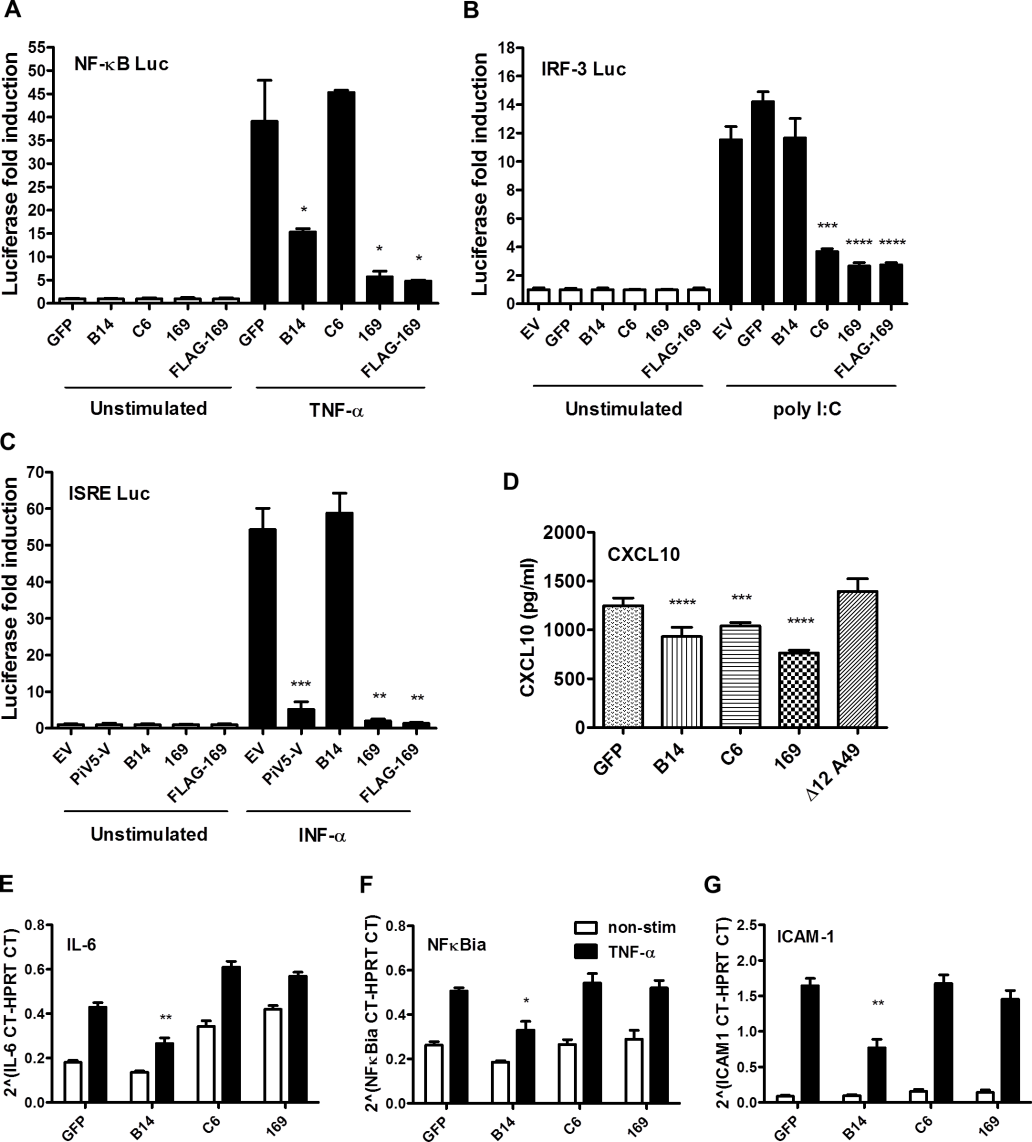
Protein 169 inhibits protein expression after activation of several innate immune signaling pathways. (**A**) HEK 293T cells were transfected in triplicate with an NF-κB reporter plasmid, TK-renilla luciferase and plasmids for expression of the indicated proteins. After 1 d the cells were stimulated with 75 ng/ml of TNF-α for 7 h or treated with the same medium lacking TNF-α. The luminescence of cell lysates was measured using a luminometer. These data are from one representative experiment (*n* = 3) and results are presented as the fold increase in luciferase expression. Firefly luciferase was normalized to renilla luciferase (internal control) and further normalized to the unstimulated samples ± SD. Statistical analysis was performed using a two-tailed Student’s t-test with Welch’s correction where necessary, * p < 0.05, ** p < 0.01, *** p < 0.001, **** p < 0.0001. (**B**) Performed as in (A) but using an ISG56.1 reporter plasmid (responsive to IRF-3) and cells were transfected with poly I:C or Lipofectamine only. (**C**) Performed as in (A) but using an ISRE reporter plasmid. The cells were stimulated with 100 U/ml of IFN-α for 7 h. (**D**) HEK 293T cells were transfected with plasmids for expression of the indicated proteins in triplicate, and the following day the cells were mock-infected or infected with SeV for 24 h. CXCL10 in the supernatant was measured by ELISA. Data shown are from one representative experiment (*n* = 2) and results are expressed as concentration of CXCL10, estimated from a nonlinear standard curve, ± SD. Statistical comparison to GFP control used a two-tailed Student’s t-test with Welch’s correction where necessary, *** p < 0.001, **** p < 0.0001. (**E, F, G**) A549 cells were transfected with plasmids for expression of the indicated proteins in triplicate and, after 24 h cells, were mock-stimulated or stimulated with 50 ng/ml of TNF-α for 7 h. Then mRNAs were extracted, cDNAs were prepared and RT-q-PCR was performed using ViiA 7 Real-Time PCR System (Life Technologies) using primers specific for IL-6 (**E**), NFκBia (**F**) and ICAM-1 (**G**). Data shown are from one representative experiment (*n* = 2) and results are expressed as cycle threshold (CT) values compared to HPRT levels ± SD. Statistical analysis was performed using a two-tailed Student’s t-test with Welch’s correction where necessary, * p < 0.05, ** p <0.01.

The *169R* gene is located in a terminal variable region of the VACV genome, is expressed early during infection and is non-essential for virus replication in cell culture. These properties are characteristic of VACV genes encoding immunevasins, such as the type I IFN binding protein [41, 42], the 3-β-hydroxysteroid dehydrogenase [43, 44] and the intracellular inhibitors of NF-κB activation [9, 10, 12–18]. Therefore, we hypothesized that protein 169 might be an immunevasin and this was tested by reporter gene assays. A plasmid in which firefly luciferase expression is driven by either an NF-κB, IRF-3 (ISG56.1), or interferon-stimulated response element (ISRE) responsive promoter was transfected separately into HEK 293T cells together with TK renilla luciferase (internal control), and plasmids expressing 169, FLAG-tagged 169 (FLAG-169) or other control proteins. The controls chosen were (i) VACV strain WR protein B14 that inhibits the NF-κB signaling by binding to IKKβ [15], (ii) VACV protein C6 that inhibits IRF-3 signaling by binding to TBK-1 adaptors [45], and (iii) paramyxovirus protein PiV5-V that inhibits type I IFN-induced signaling by degrading STAT1 [46]. Luciferase activity was measured by luminescence after stimulation with TNF-α (NF-κB Luc), IFN-α (ISRE Luc) or after transfection with poly (I:C) (IRF-3 Luc). Protein 169 and FLAG-169 inhibited NF-κB, IRF-3 and ISRE pathways as well as, or better than, known inhibitors of these pathways (Fig 4A–4C). The inhibition of all these pathways was surprising, and contrasted with the controls that generally inhibit specific pathways only. Interestingly, protein 169 also caused reduced expression of TK renilla, suggesting a general reduction in protein expression.

To investigate this further, the levels of chemokine CXCL10 were measured by ELISA. HEK 293T cells were transfected with plasmids expressing GFP, VACV B14, C6, 169 or Δ12A49 and then infected with Sendai virus (SeV). VACV protein A49 inhibits NF-κB signaling by binding to the E3 ubiquitin ligase β-TrCP but deletion of the first 12 amino acids abolishes this function [17] and so Δ12A49 served as a negative control. After 24 h, CXCL10 in the supernatant was measured by ELISA (Fig 4D). CXCL10 expression is induced by both NF-κB and IRF-3, and so levels of CXCL10 were lower in cells expressing either B14 or C6, but not in cells expressing Δ12A49, as expected. However, protein 169 also reduced CXCL10 levels, consistent with results of the reporter gene assays.

To test whether 169 mediates its inhibitory activity by blocking transcription, the levels of specific mRNAs were measured. A549 cells were transfected with plasmids expressing GFP, VACV B14, C6, or 169 and were stimulated 24 h later with TNF-α. mRNA levels of NF-κB-inducible genes such as intercellular adhesion molecule 1 (ICAM-1), IL-6, and NFκBia were measured by reverse transcription quantitative-PCR (RT-q-PCR) and normalized to the housekeeping gene hypoxanthine-guanine phosphoribosyltransferase (HPRT) (Fig 4E–4G). Levels of all three mRNAs were similar in cells expressing 169, GFP, or C6 following stimulation with TNF-α. Conversely, as expected, lower levels of these NF-κB-inducible mRNAs were detected in cells expressing the NF-κB inhibitor B14. No difference was detected in HPRT mRNA levels, confirming that the 169-mediated inhibition of multiple immune signaling pathways was not due to a general inhibition of transcription. Therefore, it was likely that protein 169 inhibited gene expression either by blocking mRNA transport to the cytoplasm, or by blocking protein synthesis. The former possibility was unlikely given that protein 169 is cytoplasmic, but was addressed by measuring the levels of cytoplasmic and nuclear mRNAs.

HEK 293T cells were co-transfected with plasmids expressing NEMO fused with renilla luciferase (NEMO-Luc) and protein 169. A plasmid expressing protein A49 and an empty vector were included as negative controls and cycloheximide was added as an inhibitor of translation. The levels of luciferase-tagged proteins were determined by luminescence (S2A Fig) and mRNA levels of NEMO-Luc were determined by RT-q-PCR (S2B Fig). In parallel, cytoplasmic and nuclear mRNAs were extracted and mRNAs levels of NEMO-Luc, HPRT and TATA box-binding protein were compared in these fractions (S2D–S2F Fig). As before, only low levels of NEMO-Luc was detected in cells expressing 169 or treated with cycloheximide. Slightly lower cytoplasmic mRNA levels of NEMO-Luc were found in cells expressing 169, but this slight decrease could not explain the profound (~10-fold) reduction of NEMO-Luc. There was also a slight reduction in NEMO-Luc mRNA in the cytoplasm in cycloheximide-treated cells, suggesting such reduction might derive from a general inhibition in protein synthesis. Lastly, no decrease in endogenous mRNAs was observed in the presence of protein 169.

Collectively these data indicate that mRNA transcription and export are not inhibited by protein 169 and therefore its inhibitory effect is downstream.

### 169 inhibits protein synthesis

**Fig 5:**
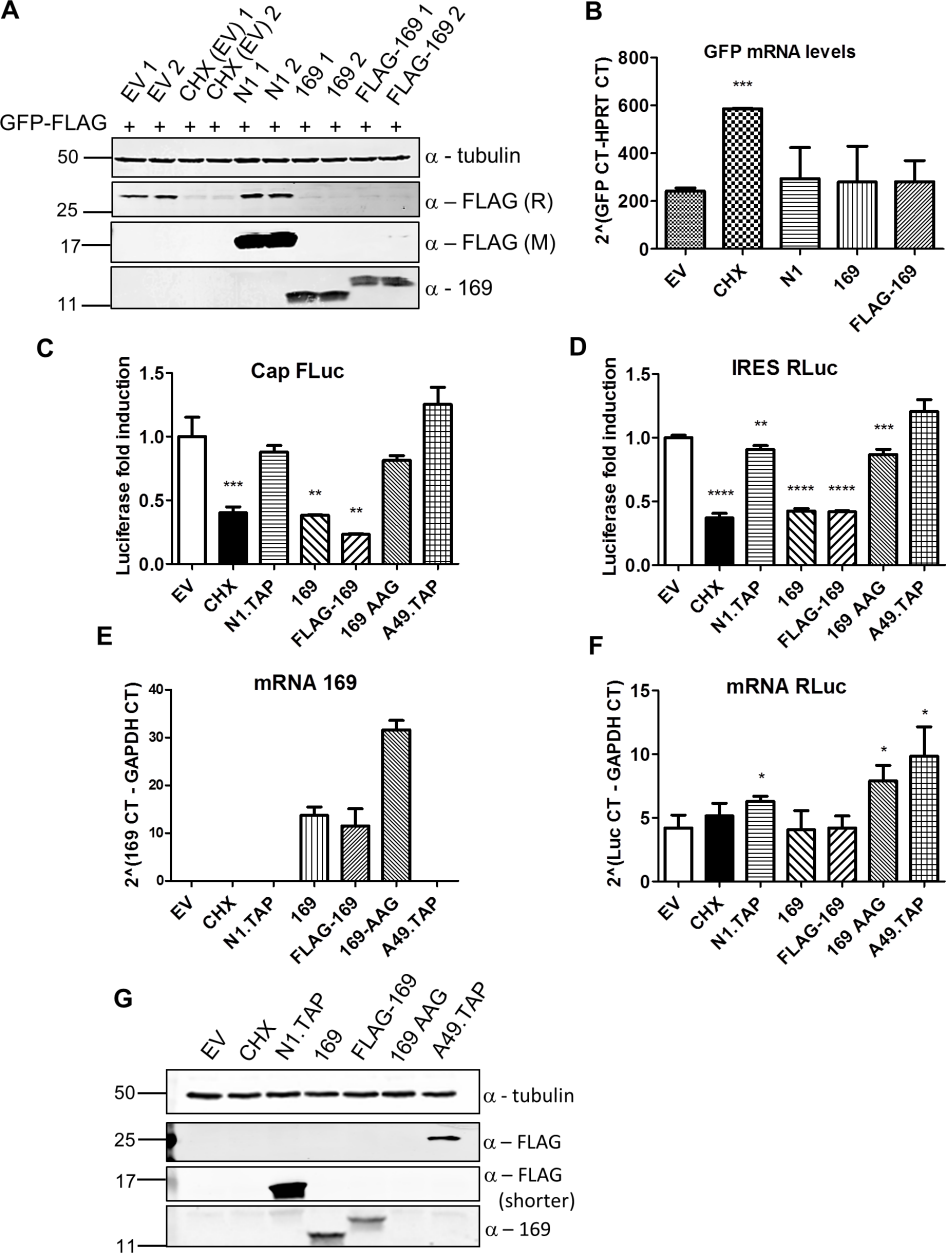
Protein 169 inhibits cap-dependent and FMDV IRES-dependent translation. (**A**) HeLa cells were transfected in duplicate with plasmids encoding the indicated proteins or with empty vector (EV) control together with a plasmid expressing GFP.FLAG. After 16 h, EV-transfected cells were treated with cycloheximide (CHX) (10 μg/ml) for 7 h. Cell lysates were resolved by SDS-PAGE and immunoblotted using antibodies against α-tubulin, 169, rabbit anti-FLAG (FLAG (R)) and mouse anti-FLAG (FLAG (M)). The positions of molecular size markers in kDa are indicated on the left. (**B**) Performed as in (A) except that mRNAs were extracted from cells, cDNAs were synthesized and GFP mRNA levels were measured by RT-q-PCR. Results are expressed as CT values compared to HPRT mRNA levels ± SD. Statistical analysis was performed using a two-tailed Student’s t-test with Welch’s correction where necessary, *** p < 0.001. Data shown are from one representative experiment (*n* = 3). (**C, D**) HEK 293T cells were transfected with a plasmid encoding a bicistronic RNA expressing firefly luciferase (FLuc, C) by a cap-dependent translation and renilla luciferase (RLuc, D) by foot and mouth disease virus (FMDV) IRES-dependent translation, together with plasmids for expression of the indicated proteins or EV control in quadruplicate. After 16 h the cells were treated with CHX (1 μg/ml) for 7 h. The relative amount of FLuc and RLuc was determined by luminescence and results are presented as the fold increase in luciferase expression normalized to the EV control ± SD. Data shown are one representative experiment (*n* = 4). (**E, F**) Performed as in (C), but in triplicate. mRNAs were extracted, cDNAs were prepared and mRNA levels of for 169 and RLuc were determined by RT-q-PCR. Results are expressed as CT values compared to GAPDH levels ± SD. Data shown are one representative experiment (*n* = 4). Statistical analysis was performed using a two-tailed Student’s t-test with Welch’s correction where necessary, ** p < 0.01, *** p < 0.01, **** p < 0.001. (**G**) Cell lysates from (A) were resolved by SDS-PAGE followed by immunoblotting with the indicated antibodies. A shorter exposure of FLAG is shown to improve visualization of N1.TAP. The positions of molecular size markers in kDa are indicated on the left.

To investigate if protein 169 inhibits protein synthesis, HeLa cells were co-transfected with plasmids expressing GFP together with VACV N1, 169, FLAG-169 or empty vector. VACV N1 is another inhibitor of NF-κB signaling [47, 48] and served as a negative control. GFP levels were determined by immunoblotting and GFP mRNAs were measured by RT-q-PCR (Fig 5A and 5B). Cycloheximide, 169 and FLAG-169 reduced GFP levels greatly compared with N1 or empty vector. In contrast, GFP mRNA levels were similar in all cells and were higher in cells treated with cycloheximide. These experiments reveal that protein 169 inhibited protein synthesis and that this is generic rather than being specific to proteins functioning in innate immunity.

VACV inhibits cap-dependent translation of host mRNAs by the de-capping enzymes D9 and D10, but these do not affect cap-independent translation [25]. To determine if protein 169 has similar or different specificity, its ability to inhibit cap-dependent and internal ribosome entry site (IRES)-dependent translation was evaluated. A plasmid encoding a bicistronic gene in which firefly luciferase is translated in a cap-dependent manner and renilla luciferase is translated in a foot and mouth disease virus (FMDV) IRES-dependent manner was transfected into HEK 293T cells together with 169, FLAG-169 or 169-AAG. The latter plasmid has the 169 initiation codon and the fourth codon mutated from AUG to AAG to prevent translation and distinguish between inhibition mediated by 169 mRNA or 169 protein. Luciferase levels were determined by luminescence (Fig 5C and 5D), mRNA levels were determined by RT-q-PCR (Fig 5E and 5F) and protein expression was also measured by immunoblotting (Fig 5G). Low levels of both firefly and renilla luciferase were found in the presence of cycloheximide, 169 and FLAG-169, but not 169 AAG, confirming that the inhibitory effect of 169 on translation requires protein 169. In contrast, luciferase levels were unaffected by proteins N1 or A49. Similar mRNA levels of renilla luciferase were found in all samples. These data show that protein 169 inhibits both cap-dependent and FMDV IRES-dependent translation.

**Fig 6:**
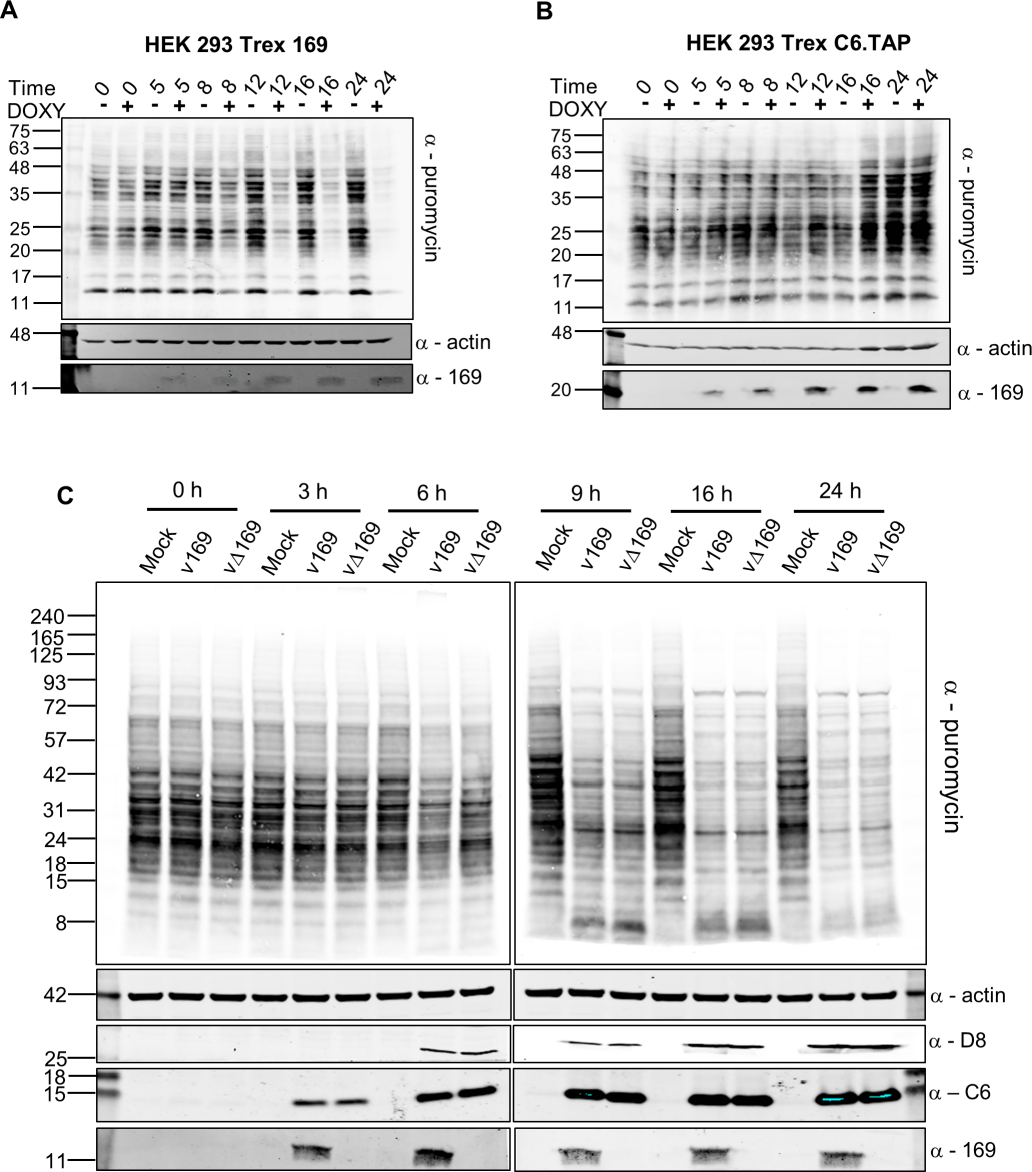
Puromycin labeling of nascent polypeptides. (**A, B**) HEK 293 Trex 169 (A) or C6.TAP (B) cells were uninduced or induced with DOXY (1 μg/ml) for the indicated times. Nascent proteins were then labeled with 5 μg/ml of puromycin for 25 min. Cell lysates were normalized by BCA assay and were resolved by SDS-PAGE and analyzed by immunoblotting using the indicated antibodies. Data shown are one representative experiment (*n* = 2). (**C**) HeLa cells were either mock-infected or infected with v169 and vΔ169 at 5 PFU/cell for the indicated times. The samples were processed as described for (A, B) above. Data shown are from one representative experiment (*n* = 2). The positions of molecular size markers in kDa are indicated on the left for all panels.

To evaluate the influence of protein 169 on protein synthesis in uninfected cells and during VACV infection, nascent proteins were analysed using surface sensing of translation (SUnSET) [49]. SUnSET is a non-radioactive method for monitoring protein synthesis that uses incorporation of puromycin into nascent polypeptide chains and causes termination of elongation. Puromycin-tagged polypeptides are then detected by immunoblotting with anti-puromycin antibody. In HEK 293 Trex cells expressing protein 169, protein synthesis was inhibited increasingly from 8 h post induction (Fig 6A). In contrast, in a control cell line expressing C6.TAP inducibly [50] no such inhibition was seen (Fig 6B).

The effect of 169 on protein synthesis during VACV infection was tested next. HeLa cells were infected with v169 or vΔ169, and puromycin was added at different times p.i. (Fig 6C). Host protein synthesis was inhibited by 6 h p.i. and more profoundly thereafter, but no difference was detected between v169 and vΔ169. This could be due to both viruses expressing the de-capping enzymes D9 and D10 that have profound effects on virus protein synthesis [25, 26, 32] and might mask effects of protein 169. This result is consistent with the observations that protein 169 is absent from virus factories (Figs 1D and 2), and does not affect virus replication and spread (Fig 3), suggesting that protein 169 might preferentially target host protein synthesis.

### 169 inhibits translation initiation

**Fig 7:**
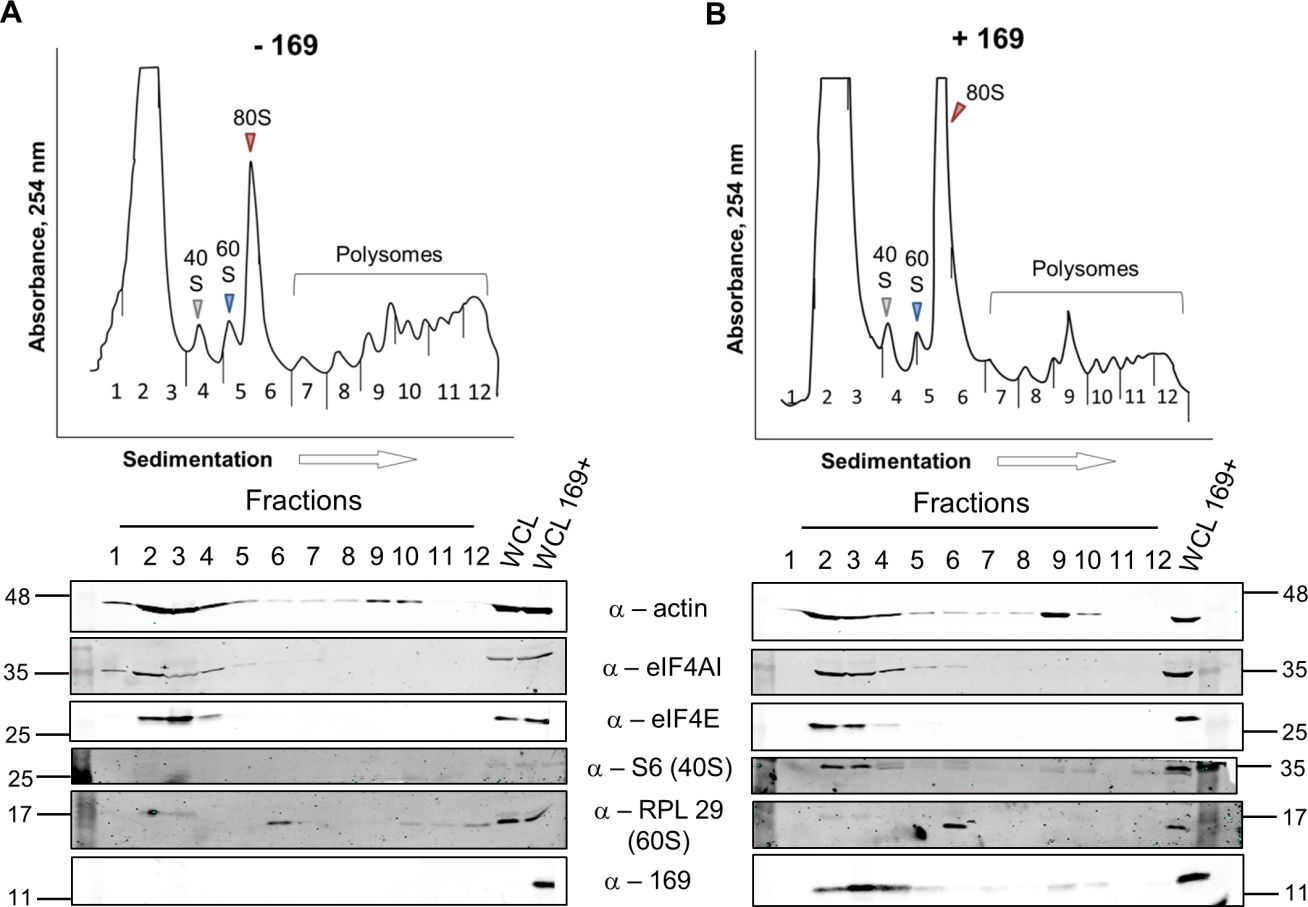
Protein 169 increases 80S monosomes and decreases polysomes. HEK 293 Trex 169 cells were uninduced (A) or induced (B) with DOXY (1 μg/ml) for 14 h. Cytoplasmic cells lysates were prepared and resolved on 10–50% sucrose density gradients (Methods). The gradient was fractionated and fractions monitored by absorbance (A_254_ nm). Indicated fractions were collected and proteins were extracted, resolved by SDS-PAGE and analyzed by immunoblotting using the indicated antibodies. The positions of molecular size markers in kDa are indicated on the left and right. WCL = whole cell lysate. Data shown are from one representative experiment (*n* = 4).

**Fig 8:**
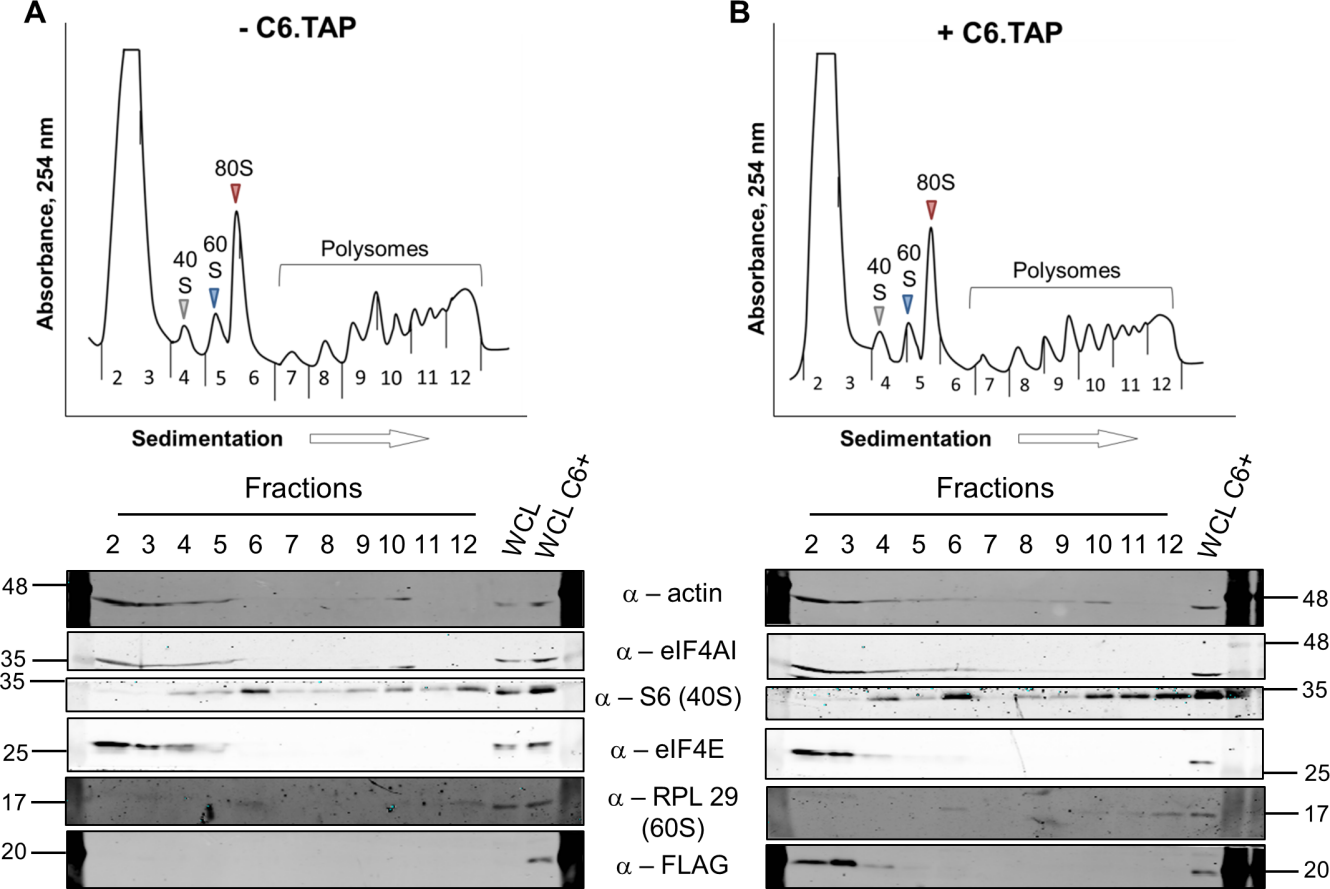
Analysis of polysomes in cells expressing protein C6. HEK 293 Trex C6.TAP cells were uninduced (A) or induced (B) with DOXY (1 μg/ml) for 14 h and then treated as described in Fig 7. Data shown are from one representative experiment (*n* = 3).

To determine at which stage of protein synthesis protein 169 might be acting, polysomes were profiled in HEK 293 Trex 169 cells with or without protein 169 expression (Fig 7A and 7B). Cytoplasmic extracts were prepared in the presence of cycloheximide to retain intact monosomes and polysomes and these were analyzed by sucrose density gradient centrifugation. The RNA and protein composition of the gradient was measured by absorbance (A_254_ nm) and immunoblotting, respectively. Protein 169 expression caused an increase in 80S ribosomes and decrease in polysomes (Fig 7B), indicating an inhibition of translational initiation. Immunoblotting of gradient fractions revealed that protein 169 co-purified partially with the 40S ribosomal fraction (Fig 7B), consistent with immunofluorescence data (Fig 2A). For comparison, HEK 293 Trex C6.TAP cells were analyzed in parallel and protein C6 expression caused no such alterations to polysomes or 80S monosomes (Fig 8A and 8B).

**Fig 9:**
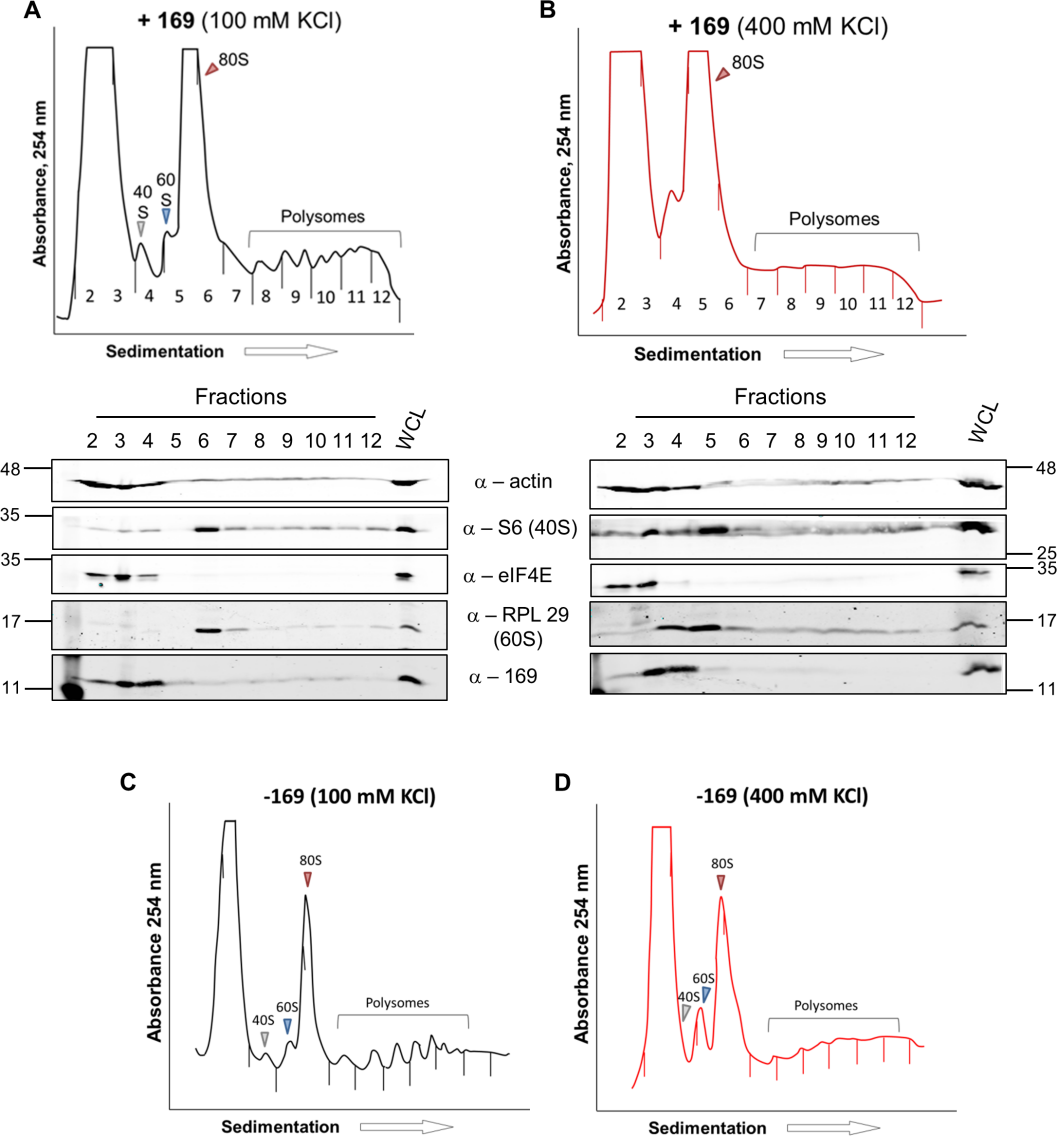
Accumulated 80S monosomes do not dissociate in high salt. **(A, B)** The experiment was performed as described in Fig 7 with HEK 293 Trex 169 cells induced with DOXY (1 μg/ml for 16 h). The cytoplasmic fraction was prepared in normal salt (100 mM KCl, A) or high salt (400 mM KCl, B) and analyzed on sucrose density gradients containing either 100 mM KCl (A) or 400 mM KCl (B). Data shown are from one representative experiment (*n* = 2). (**C**, **D**) HEK 293 Trex 169 cells were uninduced and the cytoplasmic fraction was prepared in normal salt (100 mM KCl, C) or high salt (400 mM KCl, D) and analyzed on sucrose density gradients containing either 100 mM KCl (C) or 400 mM KCl (D). Data shown are from one representative experiment (*n* = 2).

To investigate whether the 80S ribosomes accumulating in the presence of protein 169 contain mRNA, polysome profiling was repeated in a higher salt buffer (400 mM KCl), conditions in which 80S ribosomes lacking mRNA dissociate into constituent subunits. However, in the presence of protein 169, the 80S peak remained stable in high salt (Fig 9B), indicating that the 80S ribosomes are associated with mRNA. Increasing the concentration of salt in the sucrose density gradient reduced the sharpness of the peaks obtained. To confirm that this effect was due to the high salt concentration, the polysome profile of uninduced HEK 293 Trex 169 cytoplasmic cell lysates was examined on sucrose gradients (Fig 9C and 9D). Again, the high salt condition affects the overall sharpness of polysomal fractions independently of the expression of protein 169.

**Fig 10:**
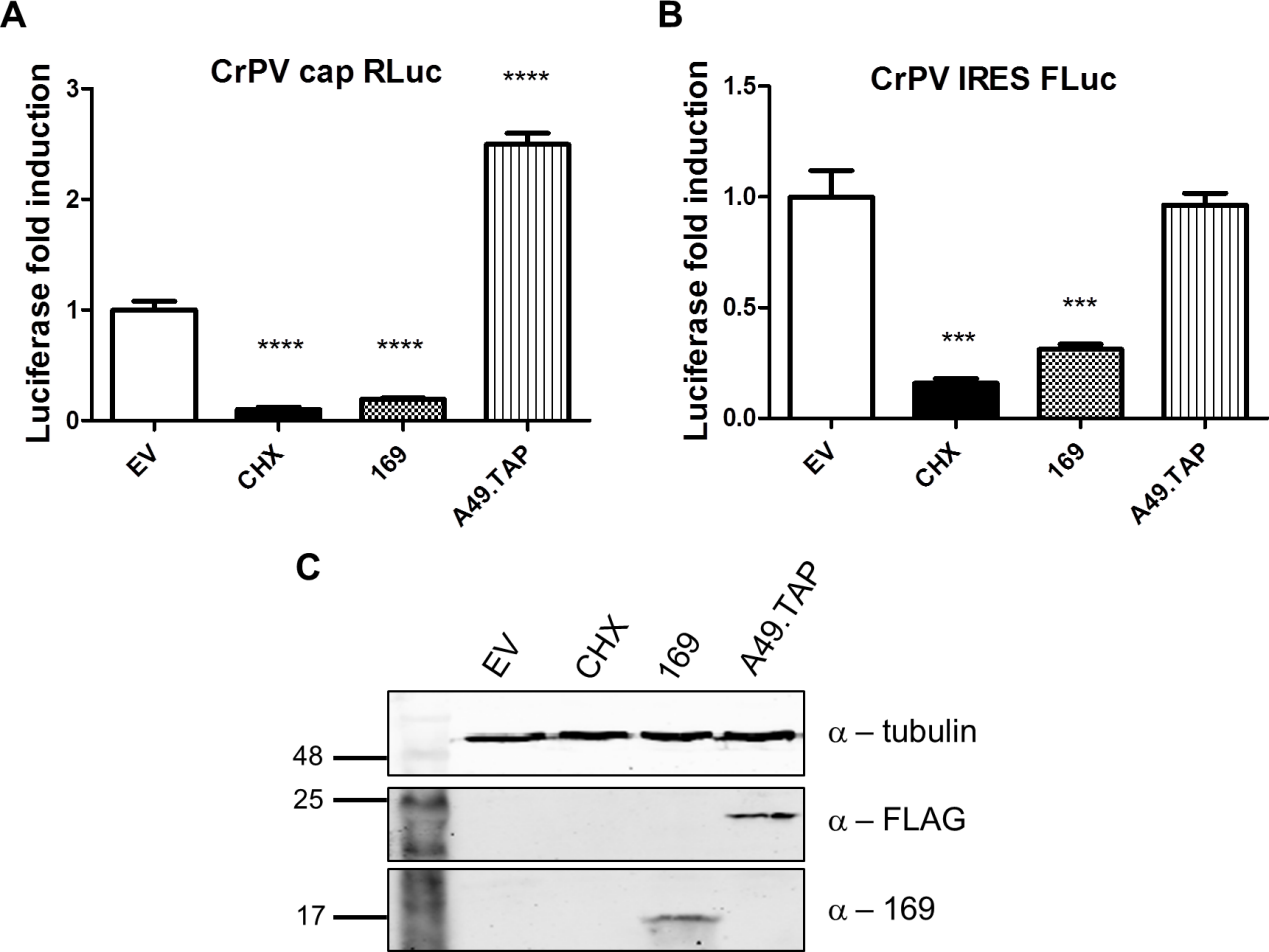
Protein 169 inhibits CrPV IRES-dependent translation. **(A, B)** HEK 293T cells were transfected with a plasmid encoding a bicistronic RNA expressing renilla luciferase (RLuc, A) by a cap-dependent translation and firefly luciferase (FLuc, B) by a CrPV IRES-dependent translation, together with plasmids for expression of A49.TAP, 169 or EV control in quintuplicate. After 7 h the cells were treated with CHX (1 μg/ml) for 16 h. The relative amount of RLuc and FLuc was determined by luminescence and results are presented as the fold increase in luciferase expression normalized to the EV control ± SD. Data shown are from one representative experiment (*n* = 5). Statistical analysis was performed using a two-tailed Student’s t-test with Welch’s correction where necessary, *** p < 0.01, **** p < 0.001. (**C**) Cell lysates from (A) were resolved by SDS-PAGE followed by immunoblotting with the indicated antibodies.

Since protein 169 inhibited cap-dependent and FMDV IRES-dependent translation, both of which require the concerted action of multiple eIFs, we tested whether protein 169 could affect translation from the cricket paralysis virus (CrPV) intergenic region (IGR) IRES. This IRES uses an unusual mechanism of translation initiation binding directly to the 40S subunit and initiating from the A-site without the requirement for initiation factors [51]. A plasmid encoding a bicistronic gene in which renilla luciferase is translated in a cap-dependent manner and firefly luciferase is translated in a CrPV IRES-dependent manner was transfected into HEK 293T cells together with A49.TAP, 169 or empty vector control. Luciferase levels were measured by luminescence (Fig 10A and 10B) and protein expression was determined by immunoblotting (Fig 10C). Low levels of both firefly and renilla luciferase were found in cycloheximide-treated cells as well as in cells expressing protein 169 indicating that protein 169 can inhibit translation from an IRES that does not require the activity of any initiation factors.

Taken together these data indicate that protein 169 inhibits the initiation of translation causing accumulation of 80S ribosomes and that this applies to both cap-dependent and IRES-dependent translation. This generic shut-down of host protein synthesis, while virus protein synthesis remains largely unaffected, affects the expression of many proteins induced by activation of innate immune sensing pathways and results in inhibition of innate immunity within infected cells. Such a strategy would be predicted to affect the outcome of infection *in vivo* and therefore this hypothesis was investigated.

### 169 modulates virus virulence

The contribution of 169 to virus virulence was examined using two murine models of infection. The intranasal (i.n.) model represents a systemic infection, where the virus replicates in the lungs and spreads to other organs. Virus virulence is assessed by measuring weight loss, virus titers and signs of illness [52, 53]. In the intradermal (i.d.) model, mice are inoculated by intradermal injection into the ear pinna, which results in a localized infection, and virulence is determined by measuring lesion size and healing time [54, 55].

**Fig 11:**
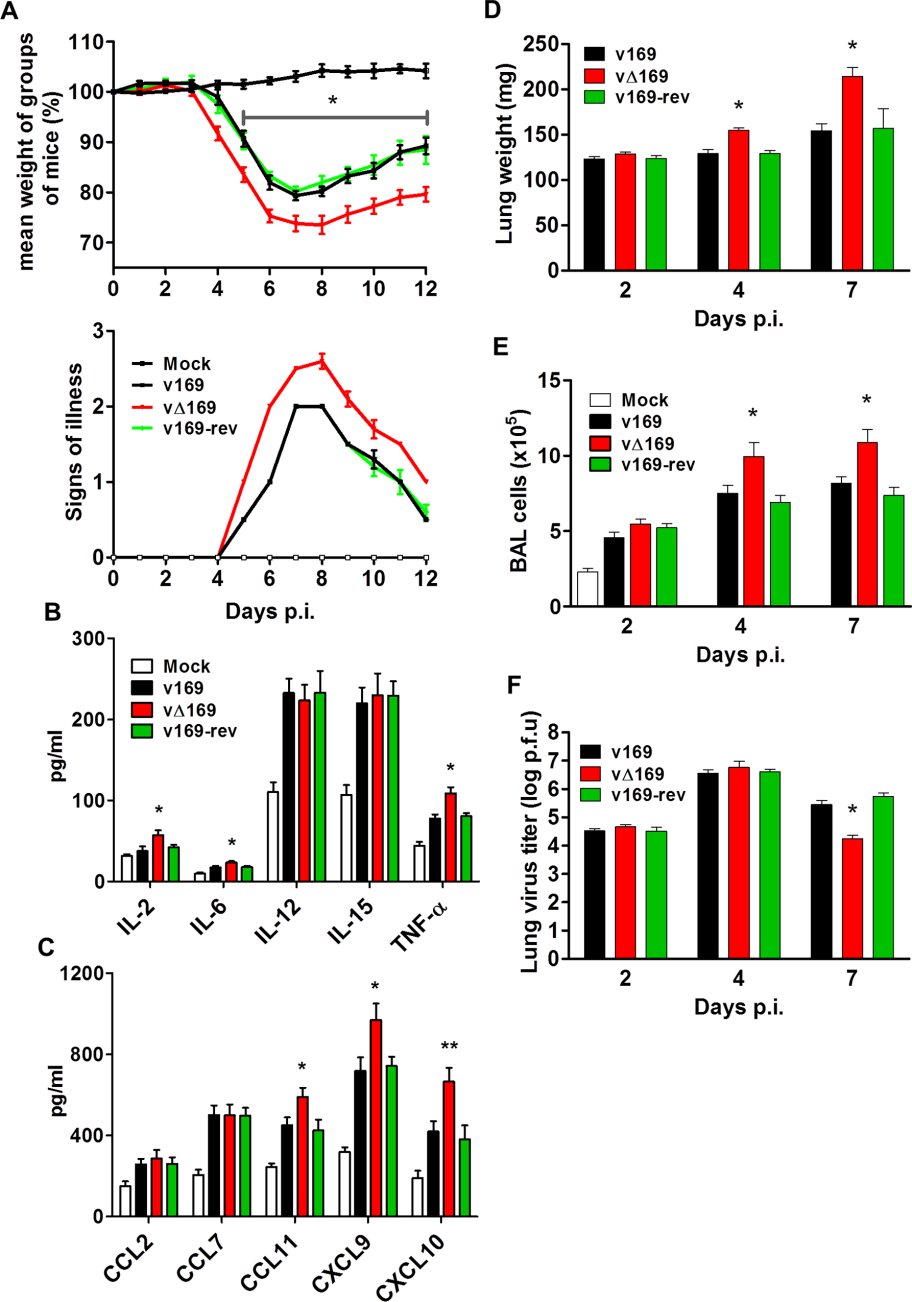
Protein 169 affects VACV virulence in the intranasal model of infection. (**A**) BALB/c mice (*n* = 5) were infected i.n. with 5 × 10^3^ PFU of the indicated purified viruses and the weight change and signs of illness were monitored daily (Methods). (**B, C**) BALB/c mice (*n* = 5) were mock treated or infected i.n. with 5 × 10^3^ PFU of the purified viruses and 24 h p.i. the mice were killed and cytokines (B) and chemokines (C) in BAL fluids were assessed by ELISA. (**D, E, F**) BALB/c mice were infected as in (A) and at the indicated times groups of mice (*n* = 5) were killed. Then the weights of the lungs were determined (D), BAL fluids were collected and the total number of cells in these fluids were counted (E), and the viral titers in lungs were determined by plaque assay (F). Data presented in (A-F) are from one representative experiment (*n* = 2) and results are expressed as the average ± SEM. Statistical analysis in all panels was performed using a two-tailed Student’s t-test, * p ≤ 0.05, ** p ≤ 0.01.

In the i.n. model, infection with vΔ169 resulted in significantly greater weight loss from day 5 onwards and more severe signs of illness than control viruses (Fig 11A). To investigate the basis for these differences, the levels of cytokines and chemokines in broncho-alveolar lavage (BAL) fluids were measured early (24 h) p.i. This showed that there were enhanced levels of IL-2, IL-6, TNF-α, CCL11, CXCL9 and CXCL10 following infection with vΔ169 compared to both control viruses, whereas the levels of CCL2, CCL9, IL-12 and IL-15 were unchanged (Fig 11B and 11C). Furthermore, infection with vΔ169 caused increased lung weights and the number of cells in BAL fluids on days 4 and 7 p.i. compared to control viruses (Fig 11D and 11E). Measurement of lung virus titers showed that all three viruses had replicated to the same extent on days 2 and 4 p.i., but by day 7 the titer of vΔ169 had decreased more than controls, indicating more rapid clearance (Fig 11F). These observations show that infection with vΔ169 caused a greater inflammatory response, with elevated synthesis of several cytokines and chemokines, enhanced recruitment of cells into BAL fluids and more rapid virus clearance.

**Fig 12:**
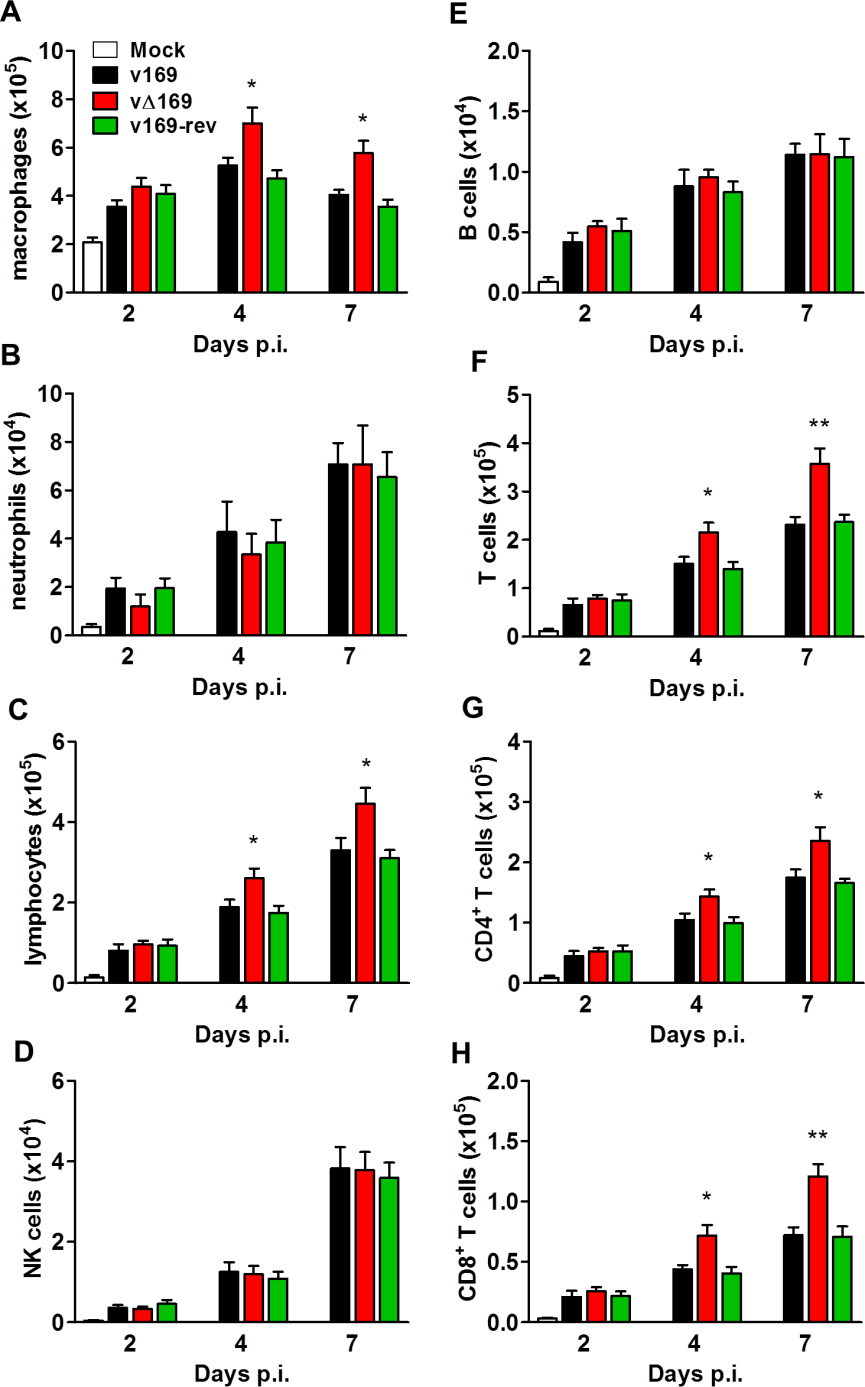
Infection with vΔ169 induces enhanced recruitment of macrophages and T-cells. (**A**) BALB/c mice (*n* = 5) were infected i.n. with 5 × 10^3^ PFU of the purified viruses and at the indicated times groups of mice (*n* = 5) were sacrificed and cells in BAL fluids were recovered, counted, and stained to determine the absolute number of macrophages (**A**), neutrophils (**B**), lymphocytes (**C**), NK cells (**D**), B cells (**E**), T cells (**F**), CD4^+^ T cells (**G**), CD8^+^ T cells (**H**) from mock-infected and VACV-infected mice by flow cytometry. Data shown are from one representative experiment (*n* = 2) and results are expressed as average ± SEM. Statistical analysis in all panels was performed using a two-tailed Student’s t-test, * p < 0.05, ** p<0.01.

To analyze the nature of cells recruited into BAL fluids, the cells were stained with monoclonal antibodies and quantified by flow cytometry. The majority of inflammatory cells recruited during infection were macrophages (Fig 12A) and lymphocytes (Fig 12C) including CD4^+^ and CD8^+^ T-cells (Fig 12F, 12G and 12H), with fewer numbers of neutrophils, (Fig 12B), NK cells (Fig 12D) and B cells (Fig 12E). Notably on days 4 and 7 p.i. the recruitment of macrophages, total lymphocytes, T-cells, and CD4^+^ and CD8^+^ T-cells was increased following infection with vΔ169 compared to controls, and these differences may explain the more rapid clearance of this virus. In contrast, neutrophils (Fig 12B), NK cells (Fig 12D), and B cells (Fig 12E) showed no difference between all viruses.

**Fig 13:**
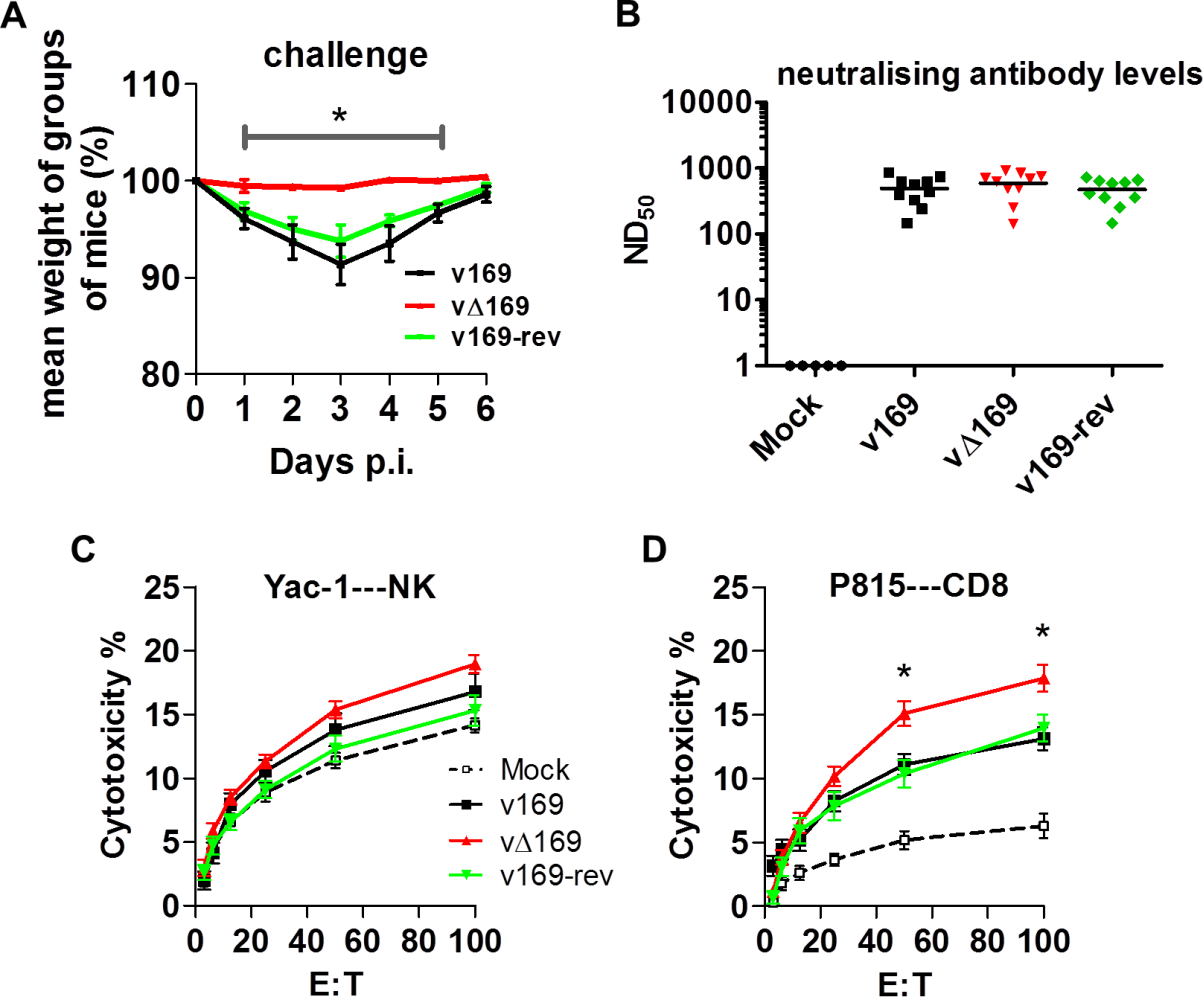
vΔ169 generates better protection after challenge. (**A**) BALB/c mice (*n* = 5) were infected i.n. with 5 × 10^3^ PFU of the purified viruses and at day 28 the mice were challenged i.n. with 5 × 10^6^ PFU of wild type virus and the weight change was determined daily. (**B**) BALB/c mice were infected as in (A) and sera were collected 28 days p.i. and assayed for neutralization of intracellular mature virus of VACV strain WR. The median value for each population is represented by a horizontal black bar. (**C, D)** BALB/c mice were infected as in (A) and at 28 days p.i. splenic lymphocytes were harvested and their ability to lyse uninfected YAC-1 (C) or VACV-infected P815 (D) cells was determined by chromium release assay. Data are presented as the percentage cell lysis at various effector to target (E:T) cell ratios. Data shown are from one representative experiment (*n* = 2) and results are expressed as the average ± SEM. Statistical analysis for all panels was performed using a two-tailed Student’s t-test, * p < 0.05.

Changes in the inflammatory response to primary infection can alter the adaptive response and subsequent protection against virus challenge. This has been observed with VACV mutants that either have increased virulence, such the VACV WR strain lacking the soluble chemokine binding protein A41 [56–58], or decreased virulence, such as the inhibitor of IRF-3 activation C6 [45, 59, 60] and the inhibitor of apoptosis and NF-κB activation N1 [47, 61, 62]. A more severe primary infection can also lead to better protection [57], and to test whether enhanced immune response generated by vΔ169 is advantageous and would lead to better protection, the potency of vΔ169 as a vaccine was evaluated. Mice were immunized via the i.n. route with v169, vΔ169 or v169-rev and then were challenged with wild type virus i.n. at day 28 (Fig 13A). In this model, vΔ169 induced better protection against challenge as shown by reduced weight loss compared to controls (Fig 13A). To investigate the basis for this, the levels of VACV neutralizing antibodies were determined by plaque reduction neutralization assay (Fig 13B) and the cytotoxicity of NK cells on uninfected YAC-1 cells (Fig 13C) and CD8^+^ splenic T-lymphocytes (Fig 13D) on VACV-infected P815 cells was measured by chromium release assay. At day 28 p.i. all groups of immunized mice had high serum antibody titers that did not differ between the groups (Fig 13B). Similarly, the cytotoxicity of splenic NK cells on YAC-1 cell targets did not differ between the groups (Fig 13C). However, the lysis of target cells by splenic CD8^+^ T-cells within the total splenocyte population from mice infected with vΔ169 was significantly greater than lysis by cells from mice infected by control viruses (Fig 13D). Collectively, these data show that immunization with vΔ169 generates stronger CD8^+^ T-cell immunological memory and better protection against challenge.

**Fig 14:**
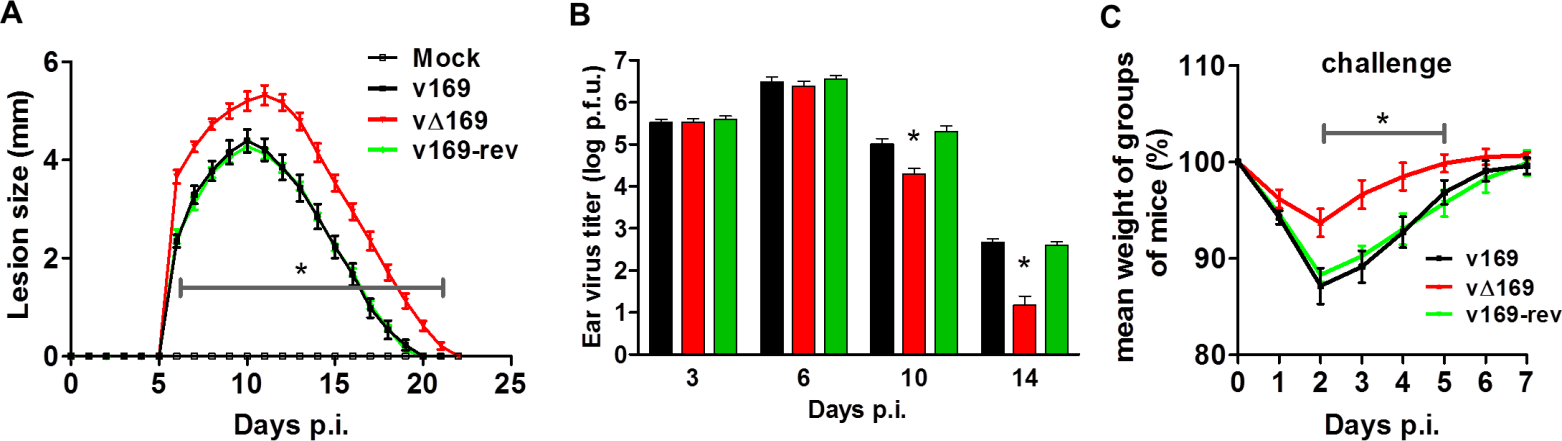
Protein 169 affects VACV virulence and immunogenicity in the intradermal model of infection. (**A**) C57BL/6 mice (*n* = 5) were infected i.d. with 1 × 10^4^ PFU in the ear pinna with purified viruses and the lesion sizes were monitored daily (Methods). (**B**) C57BL/6 mice were infected as in (A) and at the indicated times group of mice (*n* = 5) were killed and the viral titers in ears were determined by plaque assay. (**C**) C57BL/6 mice (*n* = 5) were infected i.d. with 169 recombinant viruses and after 28 days the mice were challenged i.n. with 5 × 10^6^ PFU of v169 and the weight change was determined daily. Data shown are from one representative experiment (*n* = 2) and results are presented as the average ± SEM. Statistical analysis for all panels was performed using a two-tailed Student’s t-test, * p < 0.05.

The virulence and immunogenicity of vΔ169 was also assessed after intradermal (i.d.) infection (Fig 14). vΔ169 caused a statistically significant increase in lesion size and duration compared to control viruses (Fig 14A). Furthermore, as observed in the i.n. model, viral titers in the ears showed that all viruses replicated to a similar extent initially (day 3 and 6), but thereafter (days 10 and 14) viral titers were lower for vΔ169 compared to controls (Fig 14B). Additionally, mice immunized via the i.d. route with v169, vΔ169 or v169-rev were challenged with wild type virus i.n. at day 28 (Fig 14C). As observed for i.n. model, vΔ169 induced better protection against challenge as shown by reduced weight loss of mice immunized with vΔ169 compared to controls.

## Discussion

A functional study of VACV WR protein 169 is presented. This small, highly charged protein is expressed early during VACV infection, localizes in cytoplasmic puncta but is excluded from virus factories, and inhibits the initiation of cap-dependent and cap-independent protein synthesis. Thereby, protein 169 reduces production of host inflammatory mediators induced by activation of multiple innate immune signaling pathways. Protein 169 is conserved in many VACV strains and orthopoxviruses but nonetheless is non-essential for virus replication or spread in tissue culture. Instead, it affects the outcome of infection *in vivo* by decreasing the recruitment of inflammatory leukocytes, delaying clearance of virus, reducing the memory CD8^+^ T-cell response and diminishing protection against subsequent virus challenge.

The ability of protein 169 to inhibit the innate immune response, while not affecting virus replication in cell culture or *in vivo*, is characteristic of many VACV immunevasins [8]. However, a striking difference between many of the immunevasins characterized hitherto and protein 169 is that the former are often inhibitors of an individual innate immune signaling pathway (or sometimes two pathways) by binding to one or two specific host proteins. In contrast, protein 169 is a general inhibitor of protein synthesis and targets multiple pathways that require nascent protein synthesis. Thus, by blocking the translation of host mRNAs that are transcribed, for instance, following activation of NF-κB, IRF-3 or the JAK/STAT signaling pathways, there is a decreased production of many inflammatory mediators and consequential reduced recruitment of leukocytes to the site of infection.

The inhibition of host protein synthesis by viruses is widespread, but hitherto has been considered largely a strategy by which viruses subvert host metabolism to increase virus protein synthesis and production of virions. VACV protein 169 illustrates another purpose, namely, the decrease of host protein synthesis without a concomitant increase in production of virus proteins or infectious virus particles, but with the consequence of restricting the host innate immune response to infection, so aiding virus escape and diminishing immunological memory. Protein 169 is well adapted to this purpose for it is excluded from virus factories, the site of virus protein synthesis, and so targets host translation preferentially, and its loss does not affect virus replication in cell culture or *in vivo*.

Protein 169 is also unusual in that it targets both cap-dependent and cap-independent translation. Many RNA viruses exploit IRES-dependent translation to manufacture their proteins while disabling cap-dependent translation of host mRNAs by targeting the eIF4F complex. Popular strategies are (i) cleavage of eIF4G by viral proteases [63–65], (ii) cleavage of poly A-binding protein [66, 67], and (iii) decreasing phosphorylation of cap-binding protein eIF4E [68, 69]. In contrast, DNA viruses use mostly cap-dependent translation and stimulate eIF4F formation. Herpes simplex virus type 1 (HSV-1) protein ICP0 promotes phosphorylation of eIF4E and 4E-binding protein 1 (4E-BP1) that leads to degradation of 4E-BP1 and stimulation of formation of the eIF4F complex [70]. Also, HSV-1 protein ICP6 binds eIF4G to enhance eIF4F assembly [71]. VACV stimulates eIF4F complex formation through hyper-phosphorylation of 4E-BP1 enabling interaction between eIF4E and eIF4G [27]. Protein 169 acts differently, but has some similarity with the modulation of protein synthesis by hepatitis C virus (HCV) in that it leads to alterations in innate immunity. HCV relies mainly on IRES-dependent translation and causes stimulation of protein kinase R that leads to translation inhibition through phosphorylation of eIF2α to inhibit production of IFN stimulated genes (ISGs) [72]. However, the factors responsible for these changes and their mechanism of action remain unknown.

Protein 169 is the third VACV polypeptide shown to inhibit protein synthesis, the others being the de-capping enzymes D9 and D10 [25, 32, 33]. These enzymes are made either early or late during infection and de-cap both host and viral mRNAs, although some preferential affinity for different cap structures have been shown [33]. Since the viral mRNAs are synthesized in greater abundance, these soon become predominant and so virus proteins are made while host protein synthesis declines. Rapid mRNA turnover is also important for progression between early, intermediate and late stages of VACV gene expression. The importance of de-capping for virus replication is illustrated by the loss or mutation of protein D10 that results in a smaller plaque phenotype, accumulation of early transcripts, lower virus yield [26] and attenuation *in vivo* [34]. Recently a VACV strain expressing catalytically dead versions of D9 and D10 was shown to induce large amounts of dsRNA. This activates pathways leading to inhibition of protein synthesis and consequently reduces virus production and results in severe attenuation *in vivo* [35]. In contrast, loss of protein 169 has no effect on virus replication *in vitro* (Fig 3) or *in vivo* (Figs 11 and 14) and its loss causes an increase in virulence in both i.n. and i.d. models of infection (Figs 11 and 14). In the i.n. model, infection by vΔ169 caused enhanced production of several cytokines (IL-2, IL-6 and TNF-α) and chemokines (CCL11, CXCL9 and CXCL10) within 1 day p.i. (Fig 11) and subsequent greater recruitment of macrophages and CD4^+^ and CD8^+^ T cells (Fig 12) and increased lung weight (Fig 11D). Later, this greater recruitment of inflammatory cells leads to more rapid virus clearance and recovery (Fig 11). Similarly, in the i.d. model the greater inflammatory response is reflected in a greater lesion size, but again this is followed by more rapid virus clearance and recovery (Fig 14).

The early expression of protein 169 is consistent with prior RNA analysis of the VACV genome that showed early transcription of this ORF and 169 mRNAs were detected from 1 h p.i. [73, 74]. Sometimes viruses that induce exacerbated immune responses are more virulent and in this regard it is notable that orthopoxviruses lacking ORF 169 are generally of high virulence. For instance, all sequenced variola viruses and ectromelia virus lack ORF 169 and these viruses are highly virulent in man or mice, causing smallpox and mousepox, respectively. Similarly, VACV strain Copenhagen lacks ORF 169 and caused a higher frequency of post-vaccination complications in man than the more widely used VACV strains Lister and New York City Board of Heath (Wyeth) [1]. VACV strain Copenhagen also caused larger lesion sizes in the mouse intradermal model in comparison to other VACV strains used as smallpox vaccines in man [55]. However, VACV strain Copenhagen and all variola virus strains also lack another factor that diminishes virulence, namely the soluble IL-1β binding protein encoded by gene *B15R* of VACV strain WR [53, 75], and the causes of enhanced virulence are probably multi-factorial.

The increased virulence seen by loss of gene *169R* has a few parallels in orthopoxvirus biology. In addition to deletion of the soluble IL-1β receptor encoded by VACV WR mentioned above [53], deletion of the chemokine binding protein A41 [56], and the B13 serine protease inhibitor [55] each caused an increase in virulence in either the i.n. or i.d. model, and in some cases also induced a stronger immunological memory response that resulted in better protection against virus challenge [57, 76]. Infection with vΔ169 generated a stronger innate response (Figs 11 and 12), that led to a stronger memory CD8^+^ T cell response and better protection to virus challenge (Figs 13 and 14). Increased immunological memory responses and better protection against challenge have also been observed with VACV mutants with diminished virulence, such as viruses lacking the C6 or N1 proteins [60, 77].

Protein 169 localizes mainly in the cytoplasm of infected cells throughout the course of infection. The punctate pattern observed might suggest co-localization of 169 with some specific organelles, but only some partial overlap with 40S ribosomes was observed. The precise mechanism by which protein 169 inhibits translation remains to be determined, but the polysome profiling experiments described (Figs 7–9) reveal that protein 169 expression leads to an accumulation of 80S monosomes and reduction of polysomes, particularly of heavier polysomes. This pattern is consistent with a reduced rate of translation initiation, and the stability of the 80S monosomes in high-salt indicates that the 80S ribosomes are mRNA-associated, rather than present in a free pool [78]. Reducing a pool of free ribosome is a strategy used by cardiovirus protein 2A that, in contrast to protein 169, causes accumulation of monosomes free of mRNA [79]. A direct interaction between protein 169 and either the mRNA cap or 40S subunit was not observed, nor was an effect of protein 169 on translation *in vitro* using rabbit reticulocyte lysate. However, we cannot be sure whether the prepared fraction of protein 169 is functional under the conditions tested. Nonetheless, the capacity of protein 169 to block FMDV IRES-directed translation initiation is consistent with an eIF4E-independent inhibitory mechanism. In addition, the inhibition of CrPV IRES-dependent translation by protein 169 suggests that its inhibitory activity is not mediated by interference with other eIFs.

In summary, 169 is an inhibitor of cap-dependent and cap-independent translation, it affects virus virulence and contributes to VACV immunogenicity by diminishing the innate and adaptive immune response. This study illustrates that viral inhibition of protein synthesis can be an immune evasion strategy rather than a mechanism to increase yields of virus from infected cells.

## Materials and Methods

### Ethics statement

This work was carried out in accordance with regulations of The Animals (Scientific Procedures) Act 1986. All procedures were approved by the United Kingdom Home Office and carried out under the Home Office project licence PPL 70/7116.

### Cell culture

BSC-1 (ATCC CCL-26), CV-1 (ATCC CCL70), HEK 293T (ATCC CRL-11268) and A549 (ATCC CCL-185) cells were maintained in Dulbecco’s modified minimal essential medium (DMEM) containing 10% fetal bovine serum (FBS) and penicillin/streptomycin (50 μg/ml). RK-13 (ATCC CCL-37) and TK^-^143 (ATCC CRL-8303) cells were grown in minimum essential medium (MEM) and supplemented as above. HeLa (ATCC CCL-2) cells were grown in MEM with addition of non-essential amino acids (1%) and supplemented as above. HEK 293 Trex (Invitrogen) cells were maintained in DMEM containing 10 μg/ml blasticidin, 100 μg/ml zeocin and supplemented as above.

### Plasmids

The sequence of the VACV WR *169R* gene was codon optimized by GENEART for expression in mammalian cells. *169R* was then sub-cloned into mammalian expression vectors pcDNA 3.1 or pcDNA4 TO (Invitrogen) without a tag or with an N-terminal FLAG tag. *E*. *coli* expression plasmid pOPINE were engineered to express a *169R* wild type sequence with a C-terminal His tag (169-His) and plasmid pGEX-6p-1 was engineered to express a *169R* wild type sequence with an N-terminal glutathione S-transferase (GST) tag (GST-169). Plasmid Z11-Δ169 was used to construct the VACV mutant lacking gene *169R* and contained flanking regions of the *169R* gene locus cloned into plasmid Z11 that contains the *E*. *coli* guanine phosphoribosyltransferase (*Ecogpt*) fused with enhanced green fluorescent protein (*EGFP*) driven by an early/late VACV promoter as described [45]. Plasmid Z11-169-rev was used to construct the revertant virus v169-rev and contains the *169R* gene and flanking sequences inserted into Z11 plasmid. A plasmid encoding a bicistronic gene expressing firefly luciferase in a cap-dependent manner and renilla luciferase in a FMDV IRES-dependant manner was a kind gift from Prof. Ian Goodfellow, Department of Pathology, University of Cambridge. A plasmid encoding a bicistronic reporter gene expressing firefly luciferase in a cricket paralysis virus (CrPV) IRES-dependent manner and renilla luciferase in a cap-dependant manner was a kind gift from Dr. Eric Jan, Department of Biochemistry and Molecular Biology, University of British Columbia, Canada. NF-κB-Luc, ISRE-Luc and TK renilla was obtained from Dr. Andrew Bowie (Trinity College, Dublin, Ireland), ISG56.1 Luc was from Ganeth Sen (Lerner Research Institute, Ohio), and M5P Luciferase–NEMO (Luc-NEMO) and M5P GFP-FLAG were obtained from Dr. Felix Randow (MRC Laboratory of Molecular Biology, Cambridge, United Kingdom). C6.TAP, N1.TAP, B14.FLAG and A49.TAP were described previously [15, 17, 45, 47]. V5-PiV5-V was provided by Jennifer H. Stuart (Department of Pathology, University of Cambridge, UK).

### Antibodies and reagents

Rabbit polyclonal antiserum raised against recombinant 169 protein was used for immunoblotting (diluted 1:1000–2000) and purified anti-169 770 P antibody was used for immunofluorescence (diluted 1:50). Other antibodies used were mouse anti-FLAG (Sigma, F1804, diluted 1:1000), rabbit anti-FLAG (Sigma-Aldrich, F7425, diluted 1:5000), anti-D8 mouse mAb AB1.1 against VACV protein D8 [39] (diluted 1:500), anti-α-tubulin (Millipore, 05–829, diluted 1:5000), anti-actin (Sigma, A2066, diluted 1:1000), anti-lamins A+C (Abcam, ab898, diluted 1:1000), anti-VACV protein C16 [38] (diluted 1:1000), anti-protein disulphide isomerase (PDI, 1D3 clone, Enzo Life Sciences, diluted 1:50), anti-GM130 (Transduction laboratories, diluted 1:300), anti-clathrin (Abcam, diluted 1:50), anti-human transferrin receptor (Zymed, used at 2.5 μg/ml), anti-ribosomal protein S6 (Cell Signalling, diluted 1:25 for immunofluorescence and 1:1000 for immunoblotting), anti-eIF4AI (Santa Cruz Biotechnology, N-19, diluted 1:500 dilution, a kind gift from Prof. Ian Goodfellow), anti-eIF4E (Santa Cruz Biotechnology, A-10 diluted 1:500), anti-ribosomal protein L29 (Santa Cruz Biotechnology, P-14, dilution 1:200), anti-puromycin (Millipore, clone 12D10, diluted 1:15000–1:25000), Alexa Fluor 488 goat anti-rabbit IgG (H+L) (Invitrogen, A-11008, diluted 1:750), Alexa Fluor 549 donkey anti-mouse IgG (H+L) (Invitrogen, A-10036, diluted 1:750), and MitoTracker Red CM-H2XRos (Invitrogen, M7513, diluted 1:5000). Reagents used in this study were puromycin (InvivoGen), cycloheximide (Calbiochem), doxycycline (Melford), blasticidin (Gibco) and zeocin (Invitrogen).

### Recombinant VACVs 169 construction

VACV vΔ169 was constructed by transfecting plasmid Z11-Δ169 into VACV WR infected CV-1 cells using FuGENE 6 and a recombinant VACV was isolated by transient dominant selection [40] as described for other VACV deletion mutants [12, 80]. Plaque purified wild type 169 (v169) and deletion 169 (vΔ169) viruses were isolated from the same intermediate virus and were genotyped using PCR and primers amplifying the flanking regions of the *169R* locus. The revertant 169 virus (v169-rev) was constructed by transfection of plasmid Z11-169-rev into vΔ169-infected CV-1 cells following the same procedure as described above. Genomic DNA isolated from recombinant VACVs (v169, vΔ169 and v169-rev) were compared to parental VACV WR virus using restriction endonuclease digestion with *Hin*dIII or *Sph*I digestion and virus DNA was visualized after pulsed field gel electrophoresis.

### 169 polyclonal serum production

*E*. *coli* BL21(DE3) R3 pRARE cells (kind gift from SGC Oxford), where R3 denotes a derivative of BL21(DE3) resistant to a strain of T1 bacteriophage (SGC Oxford) and the pRARE plasmid originates from the Rosetta strain (Novagen) and supplies tRNAs for rare codons, were transformed with the 169-His expression plasmid. The bacteria were grown in terrific broth and the expression of 169-His was induced by 1 mM IPTG at 37°C for 6 h. Bacteria were collected by centrifugation, lysed and disrupted by sonication. 169-His was purified from the soluble fraction by immobilized metal affinity chromatography (IMAC) using a His-Trap HP column followed by ion exchange chromatography (IEX) using a MonoQ GL column. Three and a half mg of 169-His was used to inoculate two rabbits (Eurogentec, Seraing, Belgium) to obtain polyclonal sera. Two rabbits (number 770, 771) were immunized at day 0, 14, 28 and 56 with Freund's complete adjuvant at day 0 and with incomplete Freund's adjuvant for the boosts with dose of 400 μg of 169-His. Sera prepared from venous blood drawn before immunization and at day 66 were tested for recognition of protein 169 expressed during VACV infection. Serum from rabbit 771 was sensitive enough to detect protein 169 from VACV-infected cells. This serum was used for immunoblotting analysis throughout this study (further referred as anti-169). Serum from rabbit 770 was further purified against GST-169 using AminoLink immobilization kit. GST-169 protein was produced in BL21(DE3) *E*. *coli* bacteria (Merck Millipore) transformed with pGEX-6p-1 GST-169 plasmid. Bacteria were grown in LB and expression of GST-169 was induced by 1 mM IPTG at 37°C for 6 h. Bacteria were collected by centrifugation, lysed and disrupted by sonication. GST-169 was purified from soluble fraction using glutathione-sepharose 4B and size exclusion chromatography (SEC) using Superdex 75 10/300 GL column. Two mg of GST-169 was used for polyclonal serum purification using AminoLink immobilization kit following the manufacturer’s instructions for the pH 7 protocol. Protein 169-specific purified IgG (further referred as an anti-169 purified antibody) were used for immunofluorescence studies.

### Virus growth properties

For analysis of virus single step growth properties, BSC-1 cells were infected at 10 PFU/cell for 12 or and 24 h. Extracellular virus in the clarified growth medium (after centrifugation at 500 x *g* for 10 min) was titrated by plaque assay on BSC-1 cells. Cell associated virus was measured by scraping cells from the plastic flask, combining these with the debri from the supernatant and collection by centrifugation as above. Cells were then disrupted by three rounds of freeze-thawing and sonication and the virus was titrated by plaque assay on BSC-1 cells. For analysis of multiple step growth properties, BSC-1 cells were infected at 0.05 PFU/cell for 24 and 48 h. The extracellular and cell-associated viral titers were determined as described above.

### Plaque size assay

BSC-1, RK-13 and TK^-^143 cells were infected with the indicated VACVs at 50 PFU/ well of a 6-well plate. The radius of plaques was measured after 72 h using Axiovision 4.8.2 software on an Axiovert.A1 microscope (Zeiss) with Axiocam MRc. In each condition 20 plaques per virus were measured in three independent experiments.

### Murine intranasal and intradermal models of infection

For intranasal (i.n.) model of infection, BALB/c mice (6–8 weeks old) were inoculated with VACVs, which had been purified by sedimentation twice through a sucrose cushion, (5 × 10^3^ PFU into each nostril) and monitored daily for a weight loss and scored for signs of illness as follows hair ruffling, back arching, reduced mobility, pneumonia [52, 53]. For the intradermal (i.d.) model of infection, female C57BL/6 mice (6–8 weeks old) were inoculated with purified VACVs (10^4^ PFU) in both ear pinna and the diameter of the lesion was measured daily using a micrometer [54]. The administered dose was confirmed by plaque assay. For challenge experiments, immunized animals were challenged i.n. 28 d p.i. with 5 × 10^6^ PFU of v169 and weighed daily thereafter.

Bronchial alveolar lavage (BAL) fluids were prepared on the indicated days. These were centrifuged at 1500 *g* to obtain cells for flow cytometry and the clarified supernatant was used for ELISA. Live cells collected from BAL fluids were counted using a haemocytometer following staining with trypan blue.

For determination of lung and ear tissues viral titers, the lungs and ears tissues were homogenized and washed through a 70 μm nylon mesh using DMEM and 10% FBS. Cells were then frozen and thawed three times, and sonicated thoroughly to liberate intracellular virus. Infectious virus was titrated in duplicate by plaque assay on BSC-1 cell monolayers.

For chromium-release cytotoxicity assay, NK cell cytotoxicity and VACV-specific cytotoxic T lymphocyte (CTL) activity within total splenocyte populations was assayed with a standard ^51^Cr-release assay as described [77]. NK-mediated lysis was tested on uninfected YAC-1 cells, while VACV-infected P815 cells (H-2d, mastocytoma) were used as targets for VACV-specific CTL lysis. The percentage of specific ^51^Cr release was calculated as specific lysis = [(experimental release−spontaneous release)/(total detergent release−spontaneous release)]×100. The spontaneous release values were always < 10% of total lysis.

### Antibodies, cell staining and flow cytometry

Anti-mouse CD3 (clone 145-2C11), CD4 (GK1.5), CD8 (5H10-1), CD45 (30-F11), CD45R (RA-6B2), NK1.1 (PK136), CD11b (M1/70), F4/80 (BM8), Ly-6G/Ly-6C (RB6-8C5), Ly6G (1A8) and CD16/32 (2.4G2) mAb were purchased from BD Biosciences or Biolegend. The mAbs were purified or conjugated with FITC, PerCP/cy5.5, APC, PE-Cy7, APC/Cy7, BV650 or PE. Isotype controls were used as negative controls. Flow cytometry was performed with a BD LSR Fortessa (BD Biosciences), and data were analyzed with FlowJo software (Tree Star Inc.). Events were gated for live lymphocytes on foward scatter × side scatter and dead cells were excluded on the basis of atypical fluorescence. Data were further analyzed using Prism (GraphPad, La Jolla, CA, USA).

### Cytokines and chemokines ELISA

Cytokines (IL-2, IL-6, IL-12, IL-15 and TNF-α) and chemokines (CCL2, CCL7, CCL11, CXCL9 and CXCL10) levels in the supernatants of BAL were determined following i.n. infection of 5 x 10^3^ dose in 100 μl 24 h p.i. using DuoSet ELISA kits (R&D Systems Inc.) and were carried out according to the manufacturer's instructions.

### Cell fractionation

HeLa cells were either mock-infected or infected at 10 PFU/cell for 7 h. The cells were fractionated using Cell fractionation kit (Thermo Scientific) according to the manufacturer’s instruction.

### Immunofluorescence

HeLa cells were seeded on glass coverslips and were either mock-infected of infected at 10 PFU/cell or 2 PFU/cell in case of 16 h time point. At the indicated times, the cells were washed twice with PBS and fixed with 4% paraformaldehyde in PBS containing 250 mM HEPES. The cells were permeabilized with 0.1% triton X-100 followed by blocking with 10% FBS in PBS (blocking buffer) for 0.5 h. Coverslips were incubated with primary antibodies for 1 h in a moist chamber followed by three 10 min washes with 10% FBS. Coverslips were incubated with secondary antibody (Alexa Fluor 488 Goat Anti-Rabbit IgG (H+L), Alexa Fluor 546 donkey Anti-Mouse IgG (H+L) 1:750 diluted in blocking buffer) for 30 min in a moist chamber followed by three 5 min washes with 10% FBS and PBS only. The coverslips were washed with water and mounted in Mowiol 4–88 containing DAPI. Coverslips were allowed to set and stored at 4°C. Cells were visualized by Axio observer Z1 confocal microscope (Zeiss) with a 63x oil objective.

### Reporter gene assay

Reporter gene assays was performed in HEK 293T cells in 96-well dishes as described [45]. Cells were transfected in triplicate with 60 ng of firefly reporter plasmid (NF-κB, ISG 56.1 or ISRE), 10 ng of TK renilla (as an internal control) and 100 ng of expression plasmid or empty vector control using TransIT-LT1 according to the manufacturer’s instruction. The following day cells were stimulated; (i) with 75 ng of TNF-α for 7 h (NF-κB Luc) or (ii) transfected with 200 ng/well of poly I:C for 24 h (ISG56.1 Luc) using lipofectamine, or (iii) with 100 U/ml of IFN-α for 7 h (ISRE Luc). Cells were lysed using passive lysis buffer (Promega) and firefly luciferase activity was normalized to the renilla luciferase activity, and these data were further normalized to the un-stimulated controls of each test plasmid.

### RT-q-PCR

A549 cells were transfected in triplicate with GFP.FLAG, B14.FLAG, C6.TAP and 169 using Lipofectamine LTX Plus (Life Technologies). The following day cells were stimulated with 50 ng/ml of TNF-α for 7 h. RNA was extracted using RNeasy Mini Kit (Qiagen) and converted to cDNA using SuperScript reverse transcriptase. ICAM-1, IL-6 and NF-κBia mRNA were quantified in comparison to hypoxanthine-guanine phosphoribosyltransferase (HPRT) using SYBR green master mix.

HeLa or HEK 293T cells were transfected with indicated plasmids for 24 h. RNA was extracted using RNeasy Mini Kit (Qiagen) and converted to cDNA using SuperScript reverse transcriptase. GFP, luciferase or 169 mRNA were quantified and compared to HPRT or glyceraldehyde 3-phosphate dehydrogenase (GAPDH).

For analysis of cytoplasmic and nuclear mRNA, HEK 293T cells were transfected with empty vector control, A49.TAP and 169 together with NEMO-Luc. After 4 h the cells were treated with CHX (1 μg/ml) for 16 h. Cells were lysed in RLN buffer (50 mM Tris HCl pH 8.0, 140 mM NaCl, 1.5 mM MgCl_2_, 0.5% (v/v) Nonidet P-40, 1 mM DTT, 500 U/ml RNAse out), scraped and incubated for 5 min on ice. Nuclei were sedimented by centrifugation at 1000 *g* for 3 min. Supernatant (cytoplasmic fraction) was taken and mRNA was extracted according to the manufacturer’s instruction (Qiagen). RLT buffer was added to the pellet (nuclear fraction) and forced through a 25G needle ten times. Further steps followed the manufacturer’s instructions (Qiagen). cDNA was prepared using SuperScript reverse transcriptase. Luc-NEMO, HPRT and TATA box binding protein mRNA were quantified in comparison to (GAPDH) using SYBR green master mix.

### ELISA

HEK 293T cells were transfected in triplicate with GFP.FLAG, B14.FLAG, C6.TAP, 169 and Δ12 A49.TAP. The following day cells were either mock-infected or infected with SeV for 24 h. The amount of CXCL10 in the supernatant was determined using human CXCL10 Quantikine ELISA Kit (R&D Systems). The results were analyzed using nonlinear standard curves for ELISA (GraphPad PRISM).

### HEK 293 Trex 169 inducible cell line construction

HEK 293 Trex (Invitrogen) empty cells were transfected with 169 (pcDNA4 TO) using TransIT-LT1. Transfected cells were selected in the presence of zeocin and were serially diluted to obtain individual clones. Expression of protein 169 within these clones were analyzed by immunoblotting and immunofluorescence. In the chosen clone at least 90% of cells were expressing protein 169.

### Puromycin labeling

HEK 293 Trex 169 or C6.TAP [50] cells were treated with 1 μg/ml of DOXY for the indicated times to express protein 169 or C6. Cells were treated with 5 μg/ml of puromycin for 25 min and harvested for analysis by immunoblotting [49]. HeLa cells or BSC-1 cells were mock-infected or infected with VACVs at 5 PFU/cell for the indicated times. Cells were treated with puromycin as described above.

### Polysome profiling analysis

HEK 293 Trex 169 or C6.TAP [50] cell were uninduced or induced with 1 μg/ml of DOXY for 16 h. Thirty min prior to harvesting, the cells were treated with CHX (1 μg/ml). Cells were washed and lysed in lysis buffer supplemented with protease inhibitors (cOmplete, Mini, EDTA-free, Roche, 1 tablet in 10 ml of lysis buffer) (20 mM Tris HCl pH 7.5, 100 mM KCl, 5 mM MgCl_2_, 1 mM CHX, 1 mM DTT, 0.1 mM EDTA) with DNAse I and NP-40 (0.03%) followed by trituration with a 25G needle. Cleared (19,000 *g* for 5 min at 4°C) cytoplasmic lysates were layered on top of sucrose density gradient (10–50% sucrose in lysis buffer) prepared by a Gradient Master (Biocomp) and resolved by centrifugation at 200,000 *g* for 90 min at 4°C. Absorbance (254 nm) composition within the gradient was measured during fractionation at 4°C using an Isco fractionator. Proteins from these fractions were extracted using methanol-chloroform extraction and subjected to immunoblotting analysis. Polysome profiling in higher salt condition was carried out with HEK 293 Trex 169 as described above except that the lysis buffer and sucrose density gradient contained 400 mM KCl.

### Statistical analysis

Statistical analysis was performed using Student’s two tail t-test unless otherwise stated.

## Supporting Information

S1 Fig Conservation of protein 169 in orthopoxviruses. Amino acid sequences of the predicted 169 protein from different *Orthopoxviruses* (www.poxvirus.org) were aligned using the ClustalW 2.1 programme. An asterisk (*) indicates positions at which the residue is identical in all cases, a colon (:) indicates conservation between groups of amino acid residues with strongly similar properties, a full stop (.) indicates conservation between groups of amino acid residues with weakly similar properties, and a dash (-) indicates a missing amino acid. Red color indicates different amino acid to VACV WR. CMLV–camelpox virus CMS-205, TATV–taterapox virus DAH68-168, RPXV–rabbitpox virus Utr-154, MPXV–monkeypox virus ZAR-157, CPXV–cowpox virus BR-187, ECTV–ectromelia virus CMS-206.
(TIF)

S2 Fig Protein 169 does not affect mRNA transport. (**A**) HEK 293T cells were transfected in triplicate with plasmids for expression of the indicated proteins or empty vector (EV) together with a plasmid expressing NEMO-Luc. After 4 h, EV-transfected cells were treated with CHX (1 μg/ml) for 16 h. The relative amount RLuc was determined by luminescence and the results are expressed as luciferase fold induction normalized to the EV control ± SD. (**B**) Performed as in (A) except that mRNAs were extracted, cDNAs were prepared and the level of mRNA for RLuc were determined by RT-q-PCR. Results are expressed as CT values compared to GAPDH levels ± SD. (**C**) Performed as in (A) except that the cytoplasmic fraction was prepared from lysed cells and proteins were separated and resolved by SDS-PAGE followed by immunoblotting with the indicated antibodies. The positions of molecular size markers in kDa are indicated on the left. Nuclear cell lysates prepared from cell fractionation from mock-infected HeLa cells serve as a positive control for lamin staining. (**D, E, F**) performed as in (B) except that mRNAs were extracted from cytoplasmic and nuclear fractions separately (Methods), and the mRNA levels of RLuc, HPRT and TBP were determined by RT-q-PCR. Results are expressed as CT values compared to GAPDH levels ± SD. Data shown are from one representative experiment (*n* = 3). Statistical analysis was performed using a two-tailed Student’s t-test with Welch’s correction where necessary, * p < 0.05, ** p < 0.01, *** p < 0.001, **** p < 0.0001.
(TIF)
