# Supplementary figures and images for: Inhibition of Translation Initiation by Protein 169: A Vaccinia Virus Strategy to Suppress Innate and Adaptive Immunity and Alter Virus Virulence
Source: PLoS Pathog. 2015 Sep 3;11(9):e1005151. doi: 10.1371/journal.ppat.1005151 (PMC4559412; doi:10.1371/journal.ppat.1005151)

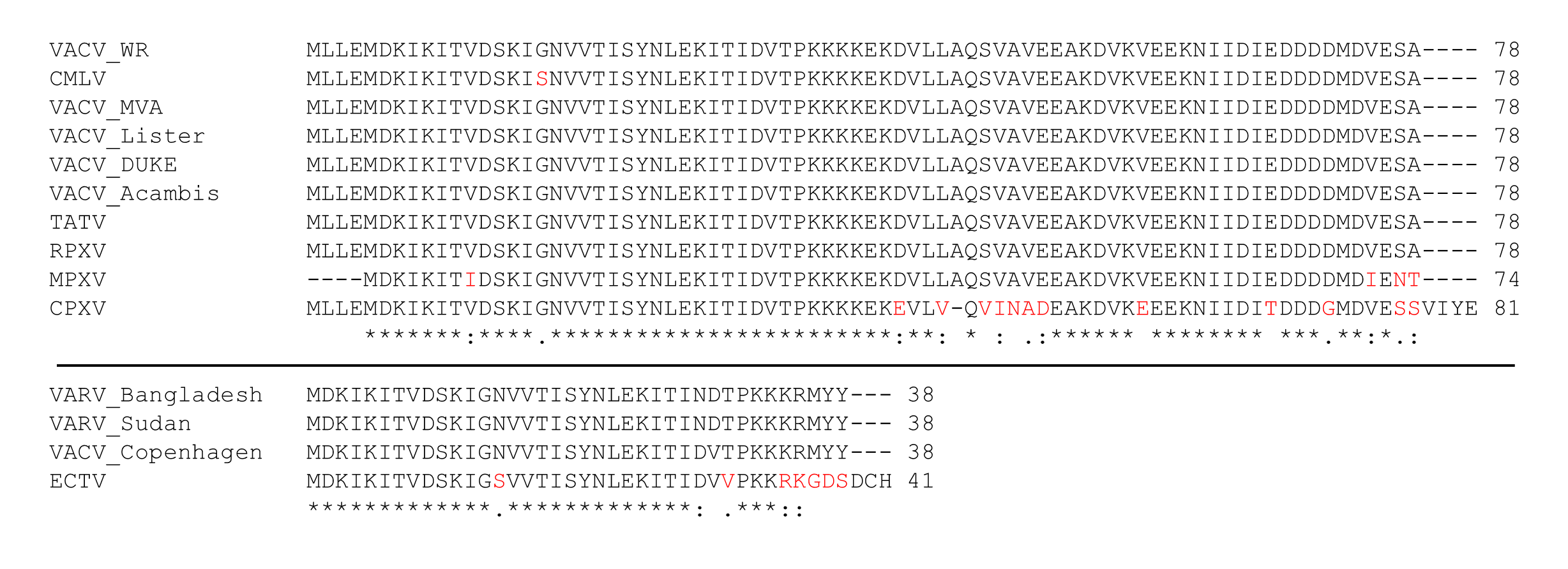

Supplement: S1 Fig — Amino acid sequences of the predicted 169 protein from different Orthopoxviruses (www.poxvirus.org) were aligned using the ClustalW 2.1 programme. An asterisk (*) indicates positions at which the residue is identical in all cases, a colon (:) indicates conservation between groups of amino acid residues with strongly similar properties, a full stop (.) indicates conservation between groups of amino acid residues with weakly similar properties, and a dash (-) indicates a missing amino acid. Red color indicates different amino acid to VACV WR. CMLV–camelpox virus CMS-205, TATV–taterapox virus DAH68-168, RPXV–rabbitpox virus Utr-154, MPXV–monkeypox virus ZAR-157, CPXV–cowpox virus BR-187, ECTV–ectromelia virus CMS-206. (TIF) [file ppat.1005151.s001.TIF]

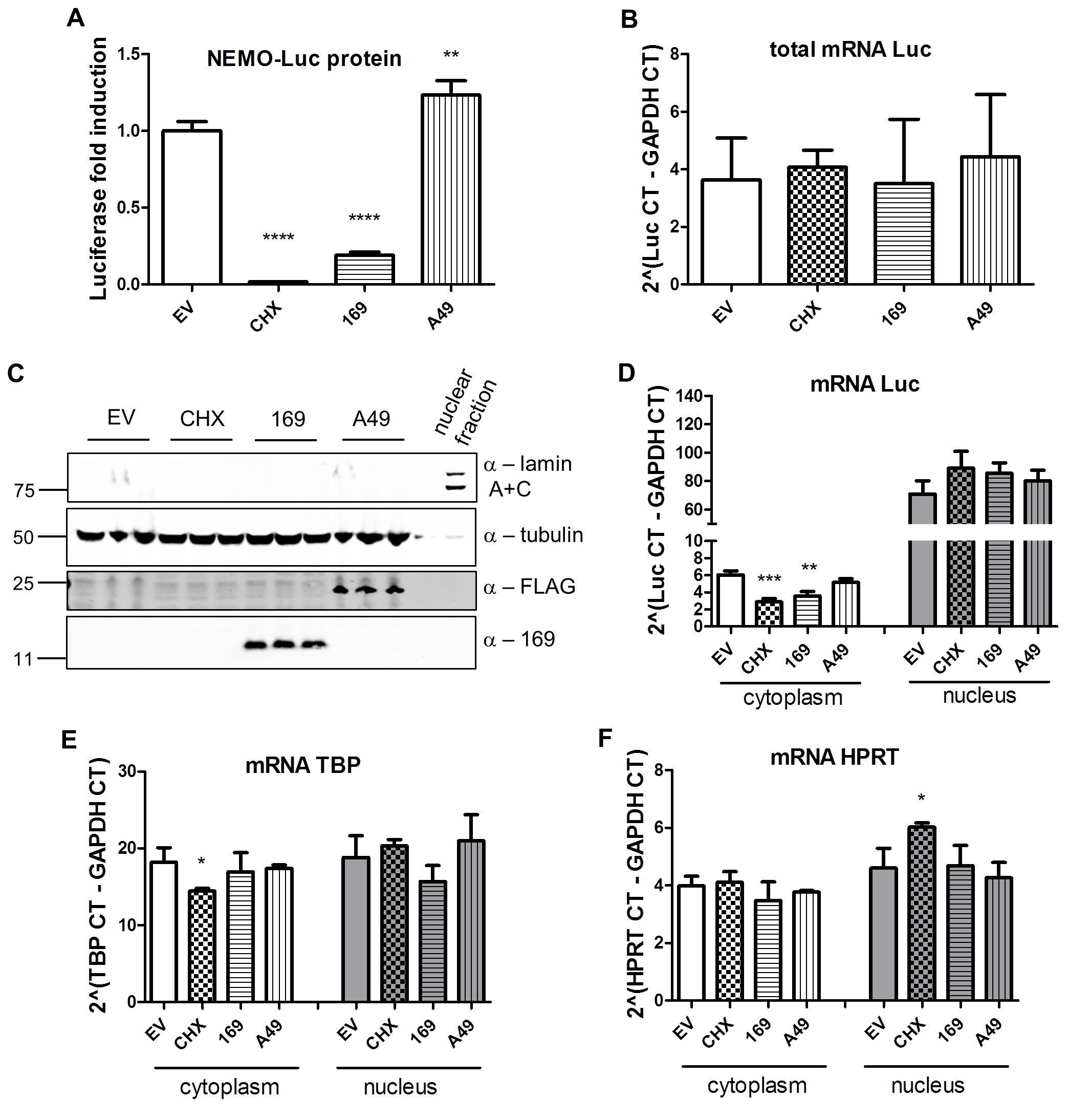

Supplement: S2 Fig — (A) HEK 293T cells were transfected in triplicate with plasmids for expression of the indicated proteins or empty vector (EV) together with a plasmid expressing NEMO-Luc. After 4 h, EV-transfected cells were treated with CHX (1 μg/ml) for 16 h. The relative amount RLuc was determined by luminescence and the results are expressed as luciferase fold induction normalized to the EV control ± SD. (B) Performed as in (A) except that mRNAs were extracted, cDNAs were prepared and the level of mRNA for RLuc were determined by RT-q-PCR. Results are expressed as CT values compared to GAPDH levels ± SD. (C) Performed as in (A) except that the cytoplasmic fraction was prepared from lysed cells and proteins were separated and resolved by SDS-PAGE followed by immunoblotting with the indicated antibodies. The positions of molecular size markers in kDa are indicated on the left. Nuclear cell lysates prepared from cell fractionation from mock-infected HeLa cells serve as a positive control for lamin staining. (D, E, F) performed as in (B) except that mRNAs were extracted from cytoplasmic and nuclear fractions separately (Methods), and the mRNA levels of RLuc, HPRT and TBP were determined by RT-q-PCR. Results are expressed as CT values compared to GAPDH levels ± SD. Data shown are from one representative experiment (n = 3). Statistical analysis was performed using a two-tailed Student’s t-test with Welch’s correction where necessary, * p < 0.05, ** p < 0.01, *** p < 0.001, **** p < 0.0001. (TIF) [file ppat.1005151.s002.TIF]
